# Deep learning–based denoising of low-dose SPECT myocardial perfusion images: quantitative assessment and clinical performance

**DOI:** 10.1007/s00259-021-05614-7

**Published:** 2021-11-15

**Authors:** Narges Aghakhan Olia, Alireza Kamali-Asl, Sanaz Hariri Tabrizi, Parham Geramifar, Peyman Sheikhzadeh, Saeed Farzanefar, Hossein Arabi, Habib Zaidi

**Affiliations:** 1 Department of Medical Radiation Engineering, Shahid Beheshti University, Tehran, Iran; 2 Research Center for Nuclear Medicine, Shariati Hospital, Tehran University of Medical Sciences, Tehran, Iran; 3 Department of Nuclear Medicine, Imam Khomeini Hospital Complex, Tehran University of Medical Sciences, Tehran, Iran; 4 Division of Nuclear Medicine and Molecular Imaging, Department of Medical Imaging, Geneva University Hospital, CH-1211 Geneva 4, Switzerland; 5 Geneva University Neurocenter, Geneva University, 1205 Geneva, Switzerland; 6 Department of Nuclear Medicine and Molecular Imaging, University of Groningen, University Medical Center Groningen, Groningen, Netherlands; 7 Department of Nuclear Medicine, University of Southern Denmark, 500 Odense, Denmark

**Keywords:** SPECT, Myocardial perfusion imaging, Denoising, Low-dose, Deep learning

## Abstract

**Purpose:**

This work was set out to investigate the feasibility of dose reduction in SPECT myocardial perfusion imaging (MPI) without sacrificing diagnostic accuracy. A deep learning approach was proposed to synthesize full-dose images from the corresponding low-dose images at different dose reduction levels in the projection space.

**Methods:**

Clinical SPECT-MPI images of 345 patients acquired on a dedicated cardiac SPECT camera in list-mode format were retrospectively employed to predict standard-dose from low-dose images at half-, quarter-, and one-eighth-dose levels. To simulate realistic low-dose projections, 50%, 25%, and 12.5% of the events were randomly selected from the list-mode data through applying binomial subsampling. A generative adversarial network was implemented to predict non-gated standard-dose SPECT images in the projection space at the different dose reduction levels. Well-established metrics, including peak signal-to-noise ratio (PSNR), root mean square error (RMSE), and structural similarity index metrics (SSIM) in addition to Pearson correlation coefficient analysis and clinical parameters derived from Cedars-Sinai software were used to quantitatively assess the predicted standard-dose images. For clinical evaluation, the quality of the predicted standard-dose images was evaluated by a nuclear medicine specialist using a seven-point (− 3 to + 3) grading scheme.

**Results:**

The highest PSNR (42.49 ± 2.37) and SSIM (0.99 ± 0.01) and the lowest RMSE (1.99 ± 0.63) were achieved at a half-dose level. Pearson correlation coefficients were 0.997 ± 0.001, 0.994 ± 0.003, and 0.987 ± 0.004 for the predicted standard-dose images at half-, quarter-, and one-eighth-dose levels, respectively. Using the standard-dose images as reference, the Bland–Altman plots sketched for the Cedars-Sinai selected parameters exhibited remarkably less bias and variance in the predicted standard-dose images compared with the low-dose images at all reduced dose levels. Overall, considering the clinical assessment performed by a nuclear medicine specialist, 100%, 80%, and 11% of the predicted standard-dose images were clinically acceptable at half-, quarter-, and one-eighth-dose levels, respectively.

**Conclusion:**

The noise was effectively suppressed by the proposed network, and the predicted standard-dose images were comparable to reference standard-dose images at half- and quarter-dose levels. However, recovery of the underlying signals/information in low-dose images beyond a quarter of the standard dose would not be feasible (due to very poor signal-to-noise ratio) which will adversely affect the clinical interpretation of the resulting images.

**Supplementary Information:**

The online version contains supplementary material available at 10.1007/s00259-021-05614-7.

## Introduction

Single-photon emission computed tomography (SPECT) is a widely used molecular imaging modality in various clinical domains, including the assessment of cardiovascular diseases [1]. SPECT myocardial perfusion imaging (MPI) is an effective non-invasive method for the diagnosis of coronary artery disease, predicting disease progression, and evaluating acute coronary artery syndromes [2, 3]. To achieve high-quality images in nuclear medicine, a sufficient dose of radiopharmaceuticals should be injected. Reducing the injected dose beyond the prescribed limit would lead to poor signal-to-noise ratio (SNR) and low-quality images, thus hampering diagnostic performance [4, 5].

Since SPECT is considered the second leading contributor to radiation dose among medical imaging modalities (with approximately 90% stress imaging studies performed annually in the USA), concerns about the radiation risks of this imaging modality have increased [6–8]. Multiple studies have been conducted to cope with the challenge of reducing the injected activity of radiopharmaceuticals in nuclear medicine imaging without sacrificing the diagnostic/clinical value. The proposed strategies fall into four categories: statistical iterative image reconstruction, post-reconstruction filtering or post-processing, recent advances in hardware, and machine learning techniques [8, 9].

Iterative image reconstruction algorithms formulate low-dose image reconstruction as a convex optimization problem and suppress noise through statistical modeling of the signal formation and noise. Advanced iterative image reconstruction algorithms have shown that the injected dose or acquisition time could be decreased by a factor of two or higher in SPECT-MPI imaging [10–14]. In this regard, Ramon et al. quantified the accuracy of perfusion-defect detection in SPECT-MPI images as a function of the injected dose to minimize the administrated dose without sacrificing diagnostic performance [12]. The other approaches rely on different post-processing and/or post-reconstruction denoising techniques, including nonlocal mean (NLM) or bilateral filters to suppress the noise in low-dose images [15–17]. Recently, innovative designs of collimators and SPECT cameras as well as novel algorithms were mainly designed to reduce scanning time or injected activity while preserving underlying information and clinical values. Scintillation crystals equipped with PMTs and parallel-hole collimators employed on conventional dual-head SPECT systems have limited performance owing to low resolution and sensitivity, and commonly require long data acquisition time, high administrated dose, etc. Dedicated cardiac SPECT instrumentation has witnessed tremendous improvements over the last few years. New dedicated commercial ultrafast solid-state cardiac cameras (DSPECT and GE 530c/570c) enable low-dose diagnostic quality imaging [8, 18–20]. In addition to the aforementioned methods, which to some extent enable the recovery of the underlying signals/structures in low-dose images, deep learning algorithms have exhibited promising performance/potential in directly estimating/predicting high-quality standard-dose images from the corresponding low-dose images [21].

It has been shown that various types of deep neural networks are capable of suppressing the noise in low-dose computed tomography (CT) as well as positron emission tomography (PET) images leading to dependable estimation of the standard-dose images [9, 22–29]. Likewise, a number of studies have been conducted in the field of low-dose SPECT-MPI. In this regard, Ramon et al. demonstrated the feasibility of using several 3D convolutional denoising networks for SPECT-MPI denoising in the image domain at 1/2, 1/4, 1/8, and 1/16 of standard clinical dose levels [30]. Song et al. investigated a 3D residual convolutional neural network (CNN) model to predict standard-dose images from 1/4-dose gated SPECT-MPI images [31]. Shiri et al. evaluated the potential of acquisition time reduction in SPECT-MPI using a residual network (ResNet) [32]. They followed two different approaches, namely, reducing the number of projections and reducing the acquisition time per projection.

The aim of this study is to reduce the administrated activity while preserving crucial/underlying structures without losing diagnostic accuracy and clinical value of SPECT-MPI images. Taking advantage of the remarkable success of deep neural networks in the field of image processing/synthesis [33], we propose an end-to-end image translation approach to denoise low-dose SPECT-MPI in the projection domain. This work employs a deep generative adversarial network (GAN) model to estimate standard-dose images from the corresponding 1/2, 1/4, and 1/8 low-dose images in an attempt to determine which reduced dose-level could be recovered by the GAN model with minimal loss of image quality and clinical value. Moreover, a comprehensive clinical assessment is conducted to assess the clinical value of deep learning–assisted prediction of standard-dose from corresponding low-dose SPECT-MPI.

## Materials and methods

### Data acquisition

This prospective single-institution study was approved by the institutional ethics committee, and all patients gave written informed consent. SPECT-MPI data were acquired for 345 patients (193 female and 152 male) scanned on the ProSPECT (Parto Negar Persia, Iran), a dedicated cardiac SPECT camera with dual-head fixed 90° angle detectors. Each head in the ProSPECT camera consists of a 40 × 25 cm^2^ thallium-activated sodium iodide (NaI(Tl)) scintillation crystal with 9.5-mm thickness and a lightweight low-energy high-resolution (LEHR) collimator with 35-mm thickness. The scintillation detector is coupled to a square array of 24 photomultiplier tubes (76 × 76 mm) which are optically connected to fused-quartz light-guide with a thickness of 20 mm. A silicon-based curing compound is employed as optical glue. Based on NEMA standards, the system spatial resolution without scatter with LEHR collimator at 10 cm from the surface of the detector, energy resolution within the useful field-of-view (UFOV), and sensitivity are 7.6 mm, 9.5%, and 79 cps/MBq, respectively [34]. To prevent radiopharmaceutical re-injection, data acquisition was carried out in list-mode format to simulate the corresponding low-dose images. Using a 2-day rest/stress acquisition protocol, image acquisition was conducted approximately 1 h after injection of 814 ± 111 MBq of ^99m^Tc-sestamibi. To reduce breast tissue and diaphragm attenuation, women and men underwent supine and prone imaging, respectively. The acquisition protocol consisted of 32 projections with 20 to 25 s per projection from the right anterior oblique (RAO) to the left posterior oblique (LPO). According to the synchronized electrocardiography (ECG) signal collected during acquisition, the detected photons were split into 8 gate intervals during a cardiac cycle.

To simulate half-dose, quarter-dose, and one-eighth-dose acquisitions, regardless of the temporal information, the number of detected photons was reduced by applying a binomial subsampling. In this subsampling method, each registered photon in the projection space would be either kept or rejected through a probability function mimicking the different low-dose levels.

The software provided with the ProSPECT camera was employed to convert the list-mode data to non-gated projection data (64 × 64 × 32 voxels) and gated projection data (64 × 64 × 256 voxels) with a voxel size of 6.4 × 6.4 × 6.4 mm^3^.

### Data preparation

Since the count rate from the liver absorption in SPECT-MPI is relatively high, projection images were manually cropped by a nuclear medicine physician to exclude the liver from cardiac images. Fifteen patients were excluded from the dataset since it was not possible to distinguish between the heart and liver. Projection data of 295 patients were randomly selected as training dataset, whereas the remaining 35 patients were used as an external test dataset to assess the performance of the GAN model. According to the clinical indication and reporting of SPECT-MPI, the patients were divided into four groups: healthy, low-risk, intermediate-risk, and severe-risk. In this light, the test dataset included 8, 16, 6, and 5 samples from these groups, respectively, to fairly evaluate the network performance.

### Deep network architecture

The GAN architecture is composed of a generator network to predict/estimate standard-dose images and a discriminator network that classifies the synthesized images as real or fake [35]. These networks are trained concurrently in an adversarial process to compete with each other. The discriminator weights are updated independently, while the generator model is updated via the discriminator feedback (Supplemental Figure 1).

#### Generator network

**Fig. 1:**
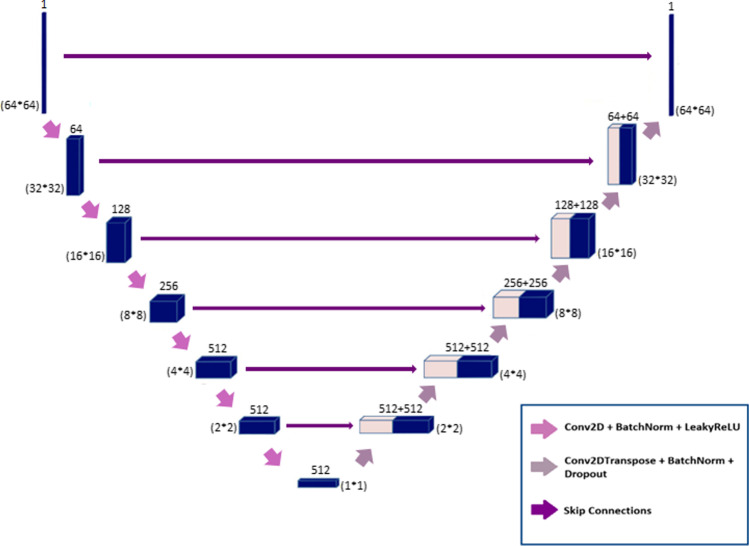
Architecture of the generator network in the GAN model

The generator network in this architecture is an encoder-decoder model (U-Net) (Fig. 1). This model utilizes low-dose images as input to estimate standard-dose images; it encodes the input image to the bottleneck layer, then decodes the data from the bottleneck layer to synthesize the output image. In this network, skip connections are used between the corresponding encoder and decoder layers.

In the encoding path, the input layer is followed by six encoder blocks. The numbers of 4 × 4 kernels with stride 2 in the encoder blocks are 64, 128, 256, 512, 512, and 512. In the second to fourth encoder blocks, the batch normalization layer is used after the convolutional layers. These layers are followed by the Leaky ReLU (with a slope of 0.2) activation function in the first five encoder blocks. Likewise, the ReLU activation function is used in the sixth encoder block.

After the bottleneck layer, six decoder blocks are used in the decoding path. In these blocks, the number of feature maps decreases from 512 to 1 according to the defined encoders. Each block consists of 4 × 4 kernel in the deconvolution layer by a stride of 2 in each direction, followed by a batch normalization layer. In the first five decoders, skip connections are used to concatenate the data from each layer in the encoder path to the corresponding layer in the decoder path. These shortcut connections are aimed to prevent the gradient vanishing issue that may occur in complicated deep neural networks. Finally, concatenated results are passed through a ReLU activation function. In the last decoder, the defined deconvolutional layer is followed by the sigmoid activation function. Empirically, in the first decoder block, we use a drop-out layer to prevent overfitting. Due to the fact that the pooling layers reduce the spatial resolution of the input images, these layers were not considered in this architecture to avoid any feature/information loss throughout the synthesis process.

The generator is updated via a weighted sum of both the adversarial loss and the L2-norm loss. The update of the trainable parameters is carried out to minimize the L2-norm loss calculated between the predicted standard-dose and the reference standard-dose images. The L2-norm loss was selected as it provided high-quality synthesis of the standard-dose SPECT images. Besides, through using adversarial loss, the generator weights are updated to minimize the loss of the discriminator (to better distinguish between real or fake samples) leading to overall better performance of the GAN model to produce more realistic images. Within the training process, a weighting factor of 100/1 was optimized in favor of the L2-norm loss, leading to overall peak performance of the GAN model.

#### Discriminator network

**Fig. 2:**
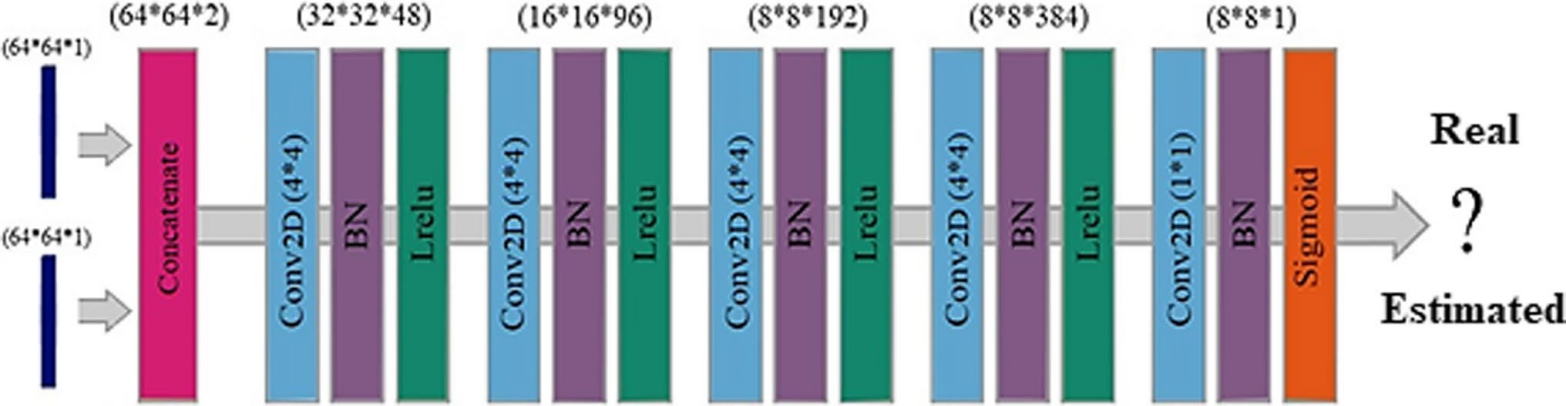
Architecture of the discriminator network in the GAN model. Conv2D, 2D convolutional layer; BN, batch normalization layer; Lrelu, Leaky ReLU activation function

The discriminator network, serving as an image classifier, takes low-dose and standard-dose images (both reference and synthesized) as inputs to determine whether the input standard-dose image is real or fake translation of the low-dose image. Figure 2 illustrates the architecture of the discriminator. The network consists of a concatenate layer and five convolutional blocks. The number of 4 × 4 kernels with stride 2 applied in the first convolutional block is 48, and this number is doubled at each three following convolutional blocks, while the stride step in the fourth convolutional block becomes 1. The 2D convolutional layer is followed by the batch normalization layer and Leaky ReLU (with a slope of 0.2) activation function in each of the four convolutional blocks. Finally, the data is passed through a 1 × 1 single-filter convolutional layer, a batch normalization layer, and a sigmoid activation function. The binary cross-entropy loss function was used for the training of the model with about 50 epochs.

The network was implemented using the Keras deep learning framework based on the TensorFlow libraries in Python 3.7. All the experiments were carried out on NVIDIA GeForce GTX 1060 with a 6 GB memory graphical processing unit. Adaptive moment estimation (Adam) optimizer with a learning rate of 0.001 was used to minimize the loss functions.

### Image reconstruction

The low-dose projection data (for 1/2, 1/4, and 1/8 levels) obtained from random sampling of the list-mode acquisition were employed for the training of the GAN model considering the standard-dose projection data as a reference. The model was trained and evaluated separately for non-gated half-dose to standard-dose, non-gated quarter-dose to standard-dose, non-gated one-eighth-dose to standard-dose, and gated half-dose to standard-dose.

Standard-dose, low-dose, and predicted standard-dose projection data from the test dataset were reconstructed using OSEM algorithm (8 iterations and 2 subsets) and the Cedars-Sinai software used to orient the images in three standard cardiac planes; short-axis (SA), vertical long-axis (VLA), and horizontal long-axis (HLA). Furthermore, we applied a post-smoothing Butterworth filter with order = 10 and cutoff = 0.45.

### Assessment strategy

#### Quantitative analysis

The quality of predicted standard-dose data, either in the projection or image space, was assessed using standard quantitative metrics, including peak signal-to-noise ratio (PSNR), root mean square error (RMSE), and structural similarity index metrics (SSIM) given in Eqs. 1, 2, and 3, respectively, considering the standard-dose data as a reference. Moreover, these metrics were also calculated for the low-dose images to provide a baseline for performance assessment of the GAN model.

1 $$\mathrm{PSNR}\left(\mathrm{dB}\right)=20 {\mathrm{log}}_{10}\left(\frac{\mathrm{Peak}}{\mathrm{MSE}}\right)$$

2 $$\mathrm{RMSE}=\sqrt{\frac{1}{n}\sum_{i=1}^{n}{({y}_{i}-\tilde{y })}^{2}}$$

3 $$\mathrm{SSIM}=\frac{(2{\mu }_{y}{\mu }_{\tilde{y }}+{C}_{1})(2{\delta }_{y.\tilde{y }}+{C}_{2})}{({\mu }_{\mathrm{y}}^{2}+{\mu }_{\tilde{y }}^{2}+{C}_{1})({\delta }_{\mathrm{y}}^{2}+{\delta }_{\tilde{y }}^{2}+{C}_{2})}$$

In Eq. (1), *Peak* indicates the maximum count of either predicted standard-dose or low-dose data, and *MSE* stands for mean squared error. In Eq. (2), *n* and *i* denote the total number of voxels and voxel index, respectively. *y* indicates the standard-dose data and *ỹ* is either the synthetic or low-dose data.${\mu }_{y}$ and ${\mu }_{\tilde{y }}$ in Eq. (3) denote the mean values of the reference and synthetic/low-dose images, respectively. ${\delta }_{\mathrm{y}.\tilde{y }}$ indicates the covariance of ${\delta }_{y}$ and ${\delta }_{\tilde{y }}$, which in turn represent the variances of the standard-dose and predicted standard-dose/low-dose images, respectively. The constant parameters *C*_1_ and *C*_2_ (*C*_1_ = 0.01 *and C*_2_ = 0.02) were set to avoid division by very small values.

#### Cedars-Sinai quantitative analysis

Extent, summed stress percent (SS%) or summed rest percent (SR%), summed stress score (SSS) or summed rest score (SRS), total perfusion deficit (TPD%), volume, wall, shape eccentricity, and shape index were calculated using quantitative perfusion SPECT (QPS) package implemented in Cedars-Sinai software. The abovementioned metrics were calculated on the reconstructed reference, low-dose, and predicted standard-dose SPECT images using the standard reconstruction settings used in clinical routine. Bland–Altman plots were sketched to describe the agreement between the predicted standard-dose/low-dose and reference standard-dose images. Finally, the Pearson correlation coefficient was computed for the derived parameters according to Eq. (4).

4 $$\rho =\frac{\sum_{i=1}^{n}({y}_{i}-{\mu }_{y})({\tilde{y }}_{i}-{\mu }_{\tilde{y }})}{\sqrt{\sum_{i=1}^{n}{({y}_{i}-{\mu }_{y})}^{2}}\sqrt{\sum_{i=1}^{n}{({\tilde{y }}_{i}-{\mu }_{\tilde{y }})}^{2}}}$$

#### Clinical evaluation

The summed score (SS) parameter was calculated for the low-dose, predicted standard-dose, and reference standard-dose reconstructed images in the test dataset by a nuclear medicine physician. Subsequently, a scoring scheme ranging from − 3 to + 3 was employed to express diagnostic differences in the predicted standard-dose/low-dose SPECT images with respect to the standard-dose ground truth, wherein 0 is equivalent to no diagnostic changes, and ± 3 is equivalent to considerable changes compared to the reference standard-dose data. Positive numbers indicate higher tracer uptake, whereas negative numbers indicate lower tracer uptake compared to the reference standard-dose images. Finally, the Pearson correlation coefficient was calculated between the reference standard-dose and the predicted standard-dose /low-dose images.

## Results

### Qualitative assessment

**Fig. 3:**
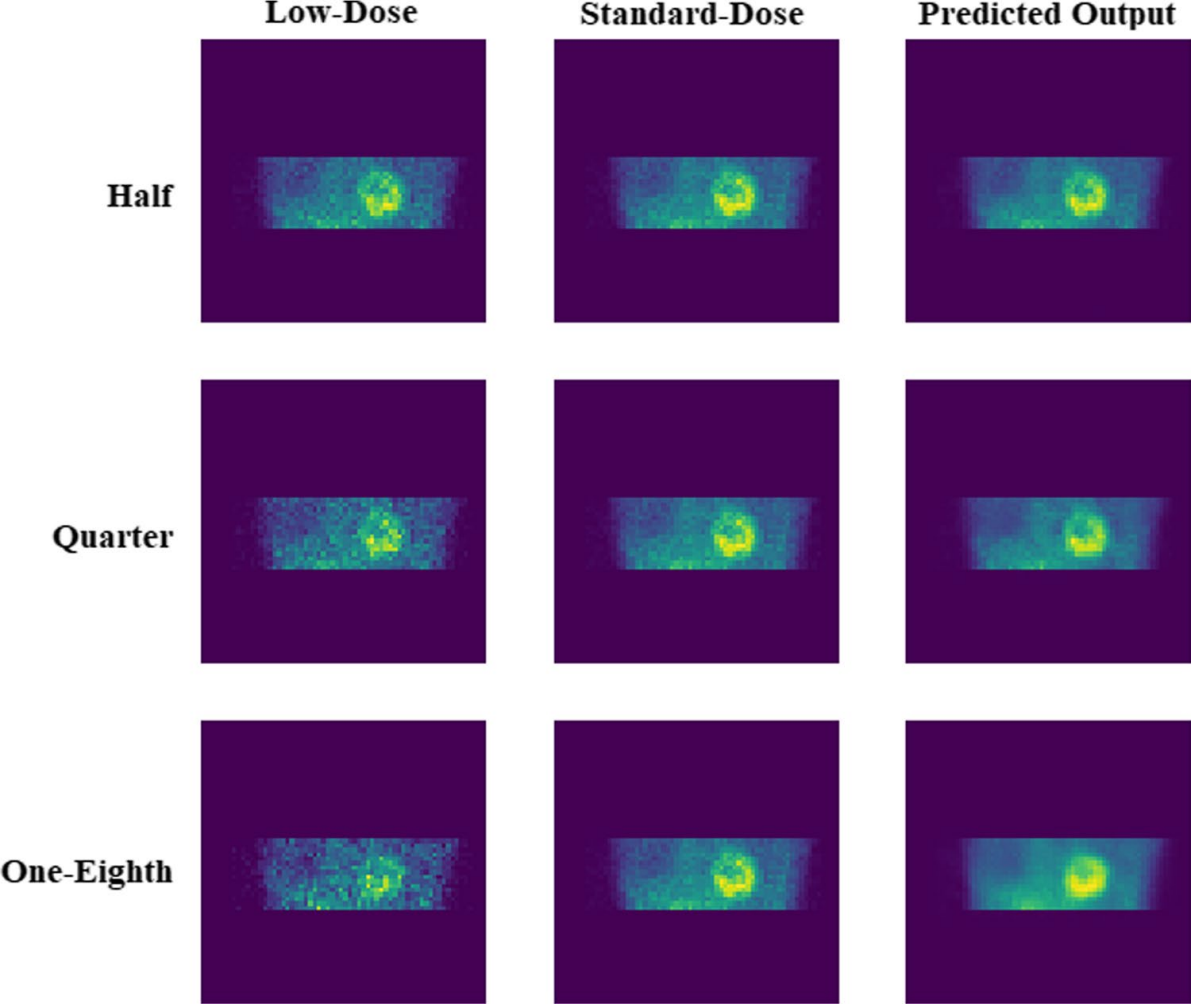
The predicted non-gated projections for a randomly selected patient from the test dataset at half-, quarter-, and one-eighth-dose levels compared to the reference standard-dose and low-dose projections

**Fig. 4:**
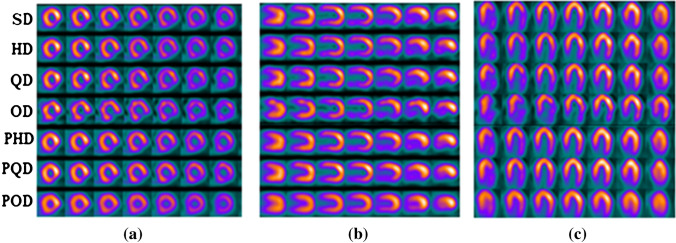
Reconstructed non-gated images for a patient with severe-risks. **a** Short-axis view, **b** long vertical-axis view, and **c** horizontal long-axis view. In **a**, **b**, and **c**, the rows from top to bottom correspond to the standard-dose (SD), half-dose (HD), quarter-dose (QD), one-eighth-dose (OD), predicted half-dose (PHD), predicted quarter-dose (PQD), and predicted one-eighth-dose (POD), respectively

The predicted standard-dose SPECT MPI in both projection and image domains exhibited considerable improvement in image quality compared to the low-dose images. Figure 3 depicts the predicted non-gated standard-dose projections for the different low-dose levels. The visual inspection revealed that at half-dose, compared to the quarter-dose and one-eighth-dose, the GAN model achieved nearly similar image quality as the reference standard-dose images. Image quality improvement is apparent for the predicted projections at quarter-dose level. However, increased signal loss is observed in the predicted projections from one-eighth-dose data. Figure 4 displays the SA, VLA, and HLA views of the reconstructed non-gated SPECT-MPI, including reference standard-dose, low-dose, and predicted standard-dose for a representative patient with severe-risk diagnosis. It can be seen that the noise is appropriately suppressed at different reduced dose levels, where the LV wall appears more uniform/natural. Overall, the predicted SPECT images exhibited good agreement with the reference standard-dose images, whereas notable signal loss and/or noise-induced pseudo-signals were observed in the low-dose images. The reconstructed non-gated images for patients diagnosed with normal perfusion, low-risk, and intermediate-risk are presented in Supplemental Figures 2-4.

### Quantitative analysis

PSNR, SSIM, and RMSE metrics were calculated separately for all 35 patients in the test dataset for the non-gated predicted and low-dose data using Eqs. (1) to (3). The mean and standard deviation of these metrics calculated in the projection and image domains are reported in Tables 1 and 2, respectively. Additionally, the paired sample *t*-test at 5% significance level was conducted between the PSNR, SSIM, and RMSE metrics obtained from the low-dose and the corresponding predicted standard-dose data. The *p* values obtained from the statistical test are also presented in Tables 1 and 2.

**Table 1:** Quantitative results associated with the different dose levels in the projection space. The *p* value between the low-dose and the predicted standard-dose projections at each reduced dose level is given

Projection space						
Parameters	HD	PHD	QD	PQD	OD	POD
**PSNR**	1.93 ± 33.22	1.33 ± 37.34	1.99 ± 28.25	1.56 ± 35.37	1.69 ± 24.51	1.77 ± 32.37
	***p* value < 0.001**		***p* value < 0.001**		***p* value < 0.001**	
**SSIM**	0.01 ± 0.96	0.01 ± 0.98	0.01 ± 0.93	0.01 ± 0.97	0.02 ± 0.89	0.01 ± 0.95
	***p* value < 0.001**		***p* value < 0.001**		***p* value < 0.001**	
**RMSE**	1.19 ± 5.69	0.52 ± 3.50	2.19 ± 10.10	0.78 ± 4.41	3.26 ± 15.52	1.23 ± 6.26
	***p* value < 0.001**		***p* value < 0.001**		***p* value < 0.001**	

*HD* half-dose, *PHD* predicted half-dose, *QD* quarter-dose, *PQD* predicted quarter-dose, *OD* one-eighth-dose, *POD* predicted one-eighth-dose

**Table 2:** Quantitative results associated with different dose levels in the image space. The *p* value between the low-dose and the predicted standard-dose projections at each reduced dose level is given

Image space						
Parameters	HD	PHD	QD	PQD	OD	POD
**PSNR**	2.68 ± 39.12	2.37 ± 42.49	2.72 ± 35.75	2.89 ± 40.17	2.74 ± 32.09	2.63 ± 33.44
	***p* value < 0.001**		***p* value < 0.001**		***p* value < 0.04**	
**SSIM**	0.01 ± 0.97	0.01 ± 0.99	0.02 ± 0.95	0.01 ± 0.98	0.04 ± 0.90	0.02 ± 0.95
	***p* value < 0.001**		***p* value < 0.001**		***p* value < 0.001**	
**RMSE**	0.96 ± 2.94	0.63 ± 1.99	1.48 ± 4.37	1.19 ± 2.93	2.04 ± 6.66	1.90 ± 5.70
	***p* value < 0.001**		***p* value < 0.001**		***p* value < 0.05**	

*HD* half-dose, *PHD* predicted half-dose, *QD* quarter-dose, *PQD* predicted quarter-dose, *OD* one-eighth-dose, *POD* predicted one-eighth-dose

There was a substantial increase in PSNR metric (12.4%, 25.2%, and 32.1%) for the predicted projections from half-, quarter-, and one-eighth-dose levels, respectively (Table 1). The SSIM increased by 2.1%, 4.3%, and 6.7%, whereas the RMSE decreased markedly by 38.5%, 56.3%, and 59.7% for the predicted projections from half-, quarter-, and one-eighth-dose levels, respectively. Table 2 summarizes the quantitative analysis results of PSNR, RMSE, and SSIM metrics in the image space. The predicted images at half-dose level achieved the highest SSIM (0.99 ± 0.01) and PSNR (42.49 ± 2.37), and the lowest RMSE (1.99 ± 0.63) with respect to the reference standard-dose images, while the predicted images at one-eighth-dose level resulted in the lowest PSNR (33.44 ± 2.63) and SSIM (0.95 ± 0.02), and the highest RMSE (5.70 ± 1.90) compared to reference standard-dose images.

**Fig. 5:**
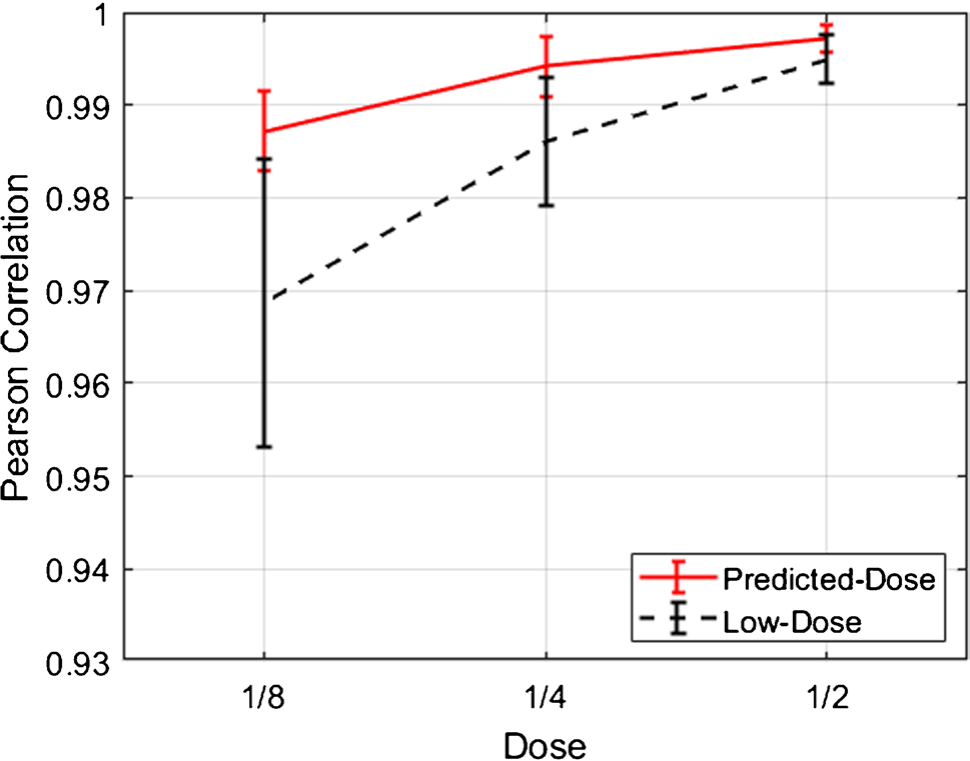
Comparison of Pearson correlation coefficients obtained from the low-dose and predicted standard-dose reconstructed images at half-, quarter-, and one-eighth-dose levels

The null hypothesis of the *t*-test was rejected in the projection and image domains for most of the cases with low *p* values. The box plots of these quantitative metrics are presented in Supplemental Figure 5. Furthermore, image quality was quantified using the Pearson correlation coefficient calculated using Eq. (4) for the low-dose and predicted standard-dose reconstructed images versus the reference standard-dose counterparts. Figure 5 shows the mean and standard deviation of the Pearson correlation coefficients obtained from 35 patients in the non-gated test dataset for all reduced dose levels. The mean of Pearson correlation coefficient increased up to 1 for the predicted images as the dose level increased from 1/8 to 1/2 wherein a significant decrease in standard deviation was observed for all dose levels compared to the corresponding low-dose images (Fig. 5). For instance, the predicted standard-dose images yielded *ρ* = 0.994 ± 0.003 compared to *ρ* = 0.987 ± 0.007 obtained from the low-dose images at quarter-dose level.

### Cedars-Sinai quantitative analysis

**Fig. 6:**
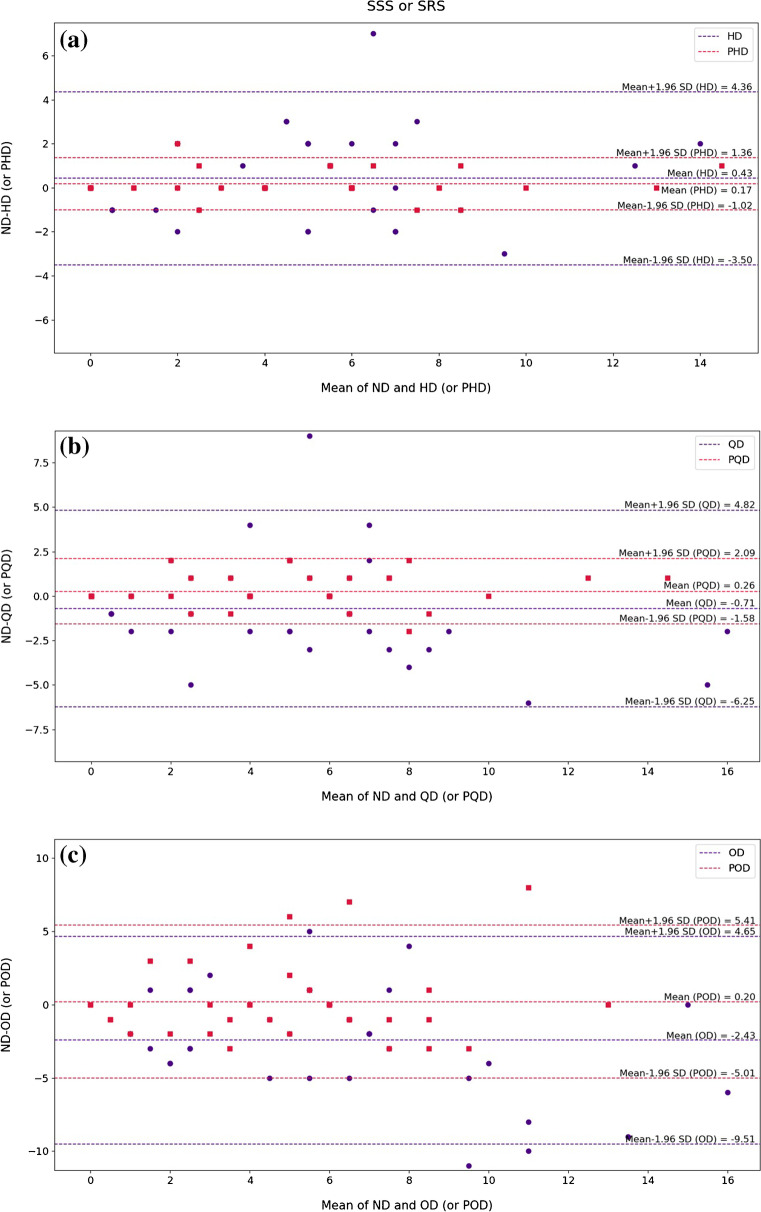
Bland–Altman plots of SSS index for the low-dose and predicted standard-dose images at **a** half-dose, **b** quarter-dose level, and **c** one-eighth-dose levels compared with the reference standard-dose images. The blue and red dashed lines indicate the mean and 95% confidence interval of the SSS differences in the low-dose and predicted standard-dose images, respectively. HD, half-dose; PHD, predicted half-dose; QD, quarter-dose; PQD, predicted quarter-dose; OD, one-eighth-dose; POD, predicted one-eighth-dose

**Fig. 7:**
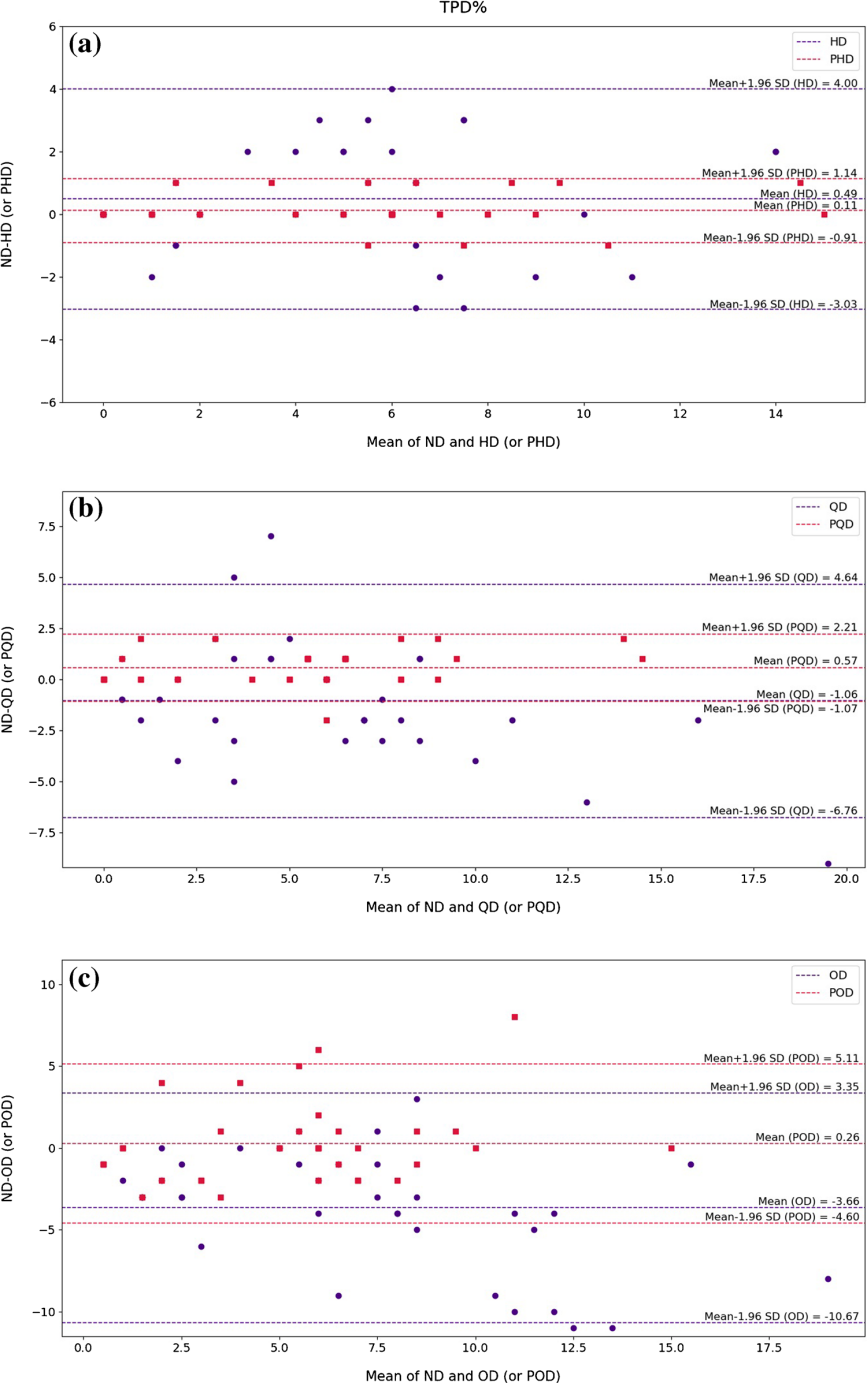
Bland–Altman plots of TPD% index for the low-dose and predicted standard-dose images at **a** half-dose, **b** quarter-dose, and **c** one-eighth-dose levels compared with the reference standard-dose images. The blue and red dashed lines designate the mean and 95% confidence interval of the TPD% differences in the low-dose and predicted standard-dose images, respectively. HD, half-dose; PHD, predicted half-dose; QD, quarter-dose; PQD, predicted quarter-dose; OD, one-eighth-dose; POD, predicted one-eighth-dose

The quantitative accuracy of the non-gated synthetic images was further investigated using QPS software indices, including Defect, Extent, SS% or SR%, SSS or SRS, TPD%, Volume, Wall, Shape Eccentricity, and Shape Index. The Bland–Altman plot was employed to present the derived indices from the low-dose and predicted standard-dose reconstructed images along with Pearson correlation analysis. The Bland–Altman plots of SSS/SRS and TPD% quantitative parameters are presented in Figs. 6 and 7 for the different dose levels. Considering the SSS/SRS index shown in Fig. 6, the Bland–Altman plots displayed the lowest bias (0.17) and variance (95% CI: − 1.02, + 1.36) for the predicted half-dose images compared with the reference standard-dose images. At quarter-dose level, the data points associated with the low-dose images exhibited variability nearly twice as large as the predicted standard-dose images. At one-eighth-dose level, despite the large dispersion of the data points, closer agreement was observed between the predicted standard-dose and reference standard-dose images in comparison with the low-dose images. Likewise, the TPD% index presented in Fig. 7 showed less bias and variance in the predicted standard-dose images than the low-dose images at all reduced dose levels. The Bland–Altman plots for the rest of the indices can be found in Supplemental Figures 6–12. Moreover, the box plots of these indices at the different dose levels are shown in Supplemental Fig. 13.

Table 3 presents the Pearson correlation coefficients for the QPS indices. Despite the overall improvements seen for the predicted half-dose and quarter-dose images, no significant improvement was observed for the one-eighth-dose level due to the extremely high noise level.

**Table 3:** Pearson correlation coefficients of the QPS quantitative parameters considering the actual standard-dose images as reference

Parameters	HD	PHD	QD	PQD	OD	POD
**Defect**	0.94	0.99	0.90	0.99	0.83	0.75
	***p* value < 0.001**		***p* value < 0.001**		***p* value < 0.03**	
**Extent**	0.87	0.99	0.83	0.97	0.71	0.70
	***p* value < 0.001**		***p* value < 0.001**		***p* value < 0.01**	
**SSS / SRS**	0.83	0.99	0.75	0.96	0.68	0.70
	***p* value < 0.001**		***p* value < 0.001**		***p* value < 0.01**	
**SS% / SR%**	0.82	0.99	0.75	0.97	0.66	0.70
	***p* value < 0.001**		***p* value < 0.001**		***p* value < 0.01**	
**TPD%**	0.88	0.99	0.82	0.98	0.76	0.76
	***p* value < 0.001**		***p* value < 0.001**		***p* value < 0.01**	
**Volume**	0.99	1.00	0.99	1.00	0.95	0.97
	***p* value < 0.001**		***p* value < 0.03**		***p* value = 0.29**	
**Wall**	0.98	1.00	0.97	1.00	0.93	0.96
	***p* value < 0.001**		***p* value < 0.001**		***p* value = 0.39**	
**Shape Eccentricity**	0.94	0.99	0.93	0.99	0.84	0.82
	***p* value < 0.001**		***p* value < 0.001**		***p* value < 0.01**	
**Shape Index**	0.72	0.95	0.60	0.88	0.54	0.72
	***p* value < 0.001**		***p* value < 0.004**		***p* value < 0.03**	

*HD* half-dose, *PHD* predicted half-dose, *QD* quarter-dose, *PQD* predicted quarter-dose, *OD* one-eighth-dose, *POD* predicted one-eighth-dose. *p* value between the low-dose and the predicted standard-dose projections at each reduced dose level

### Clinical evaluation

**Fig. 8:**
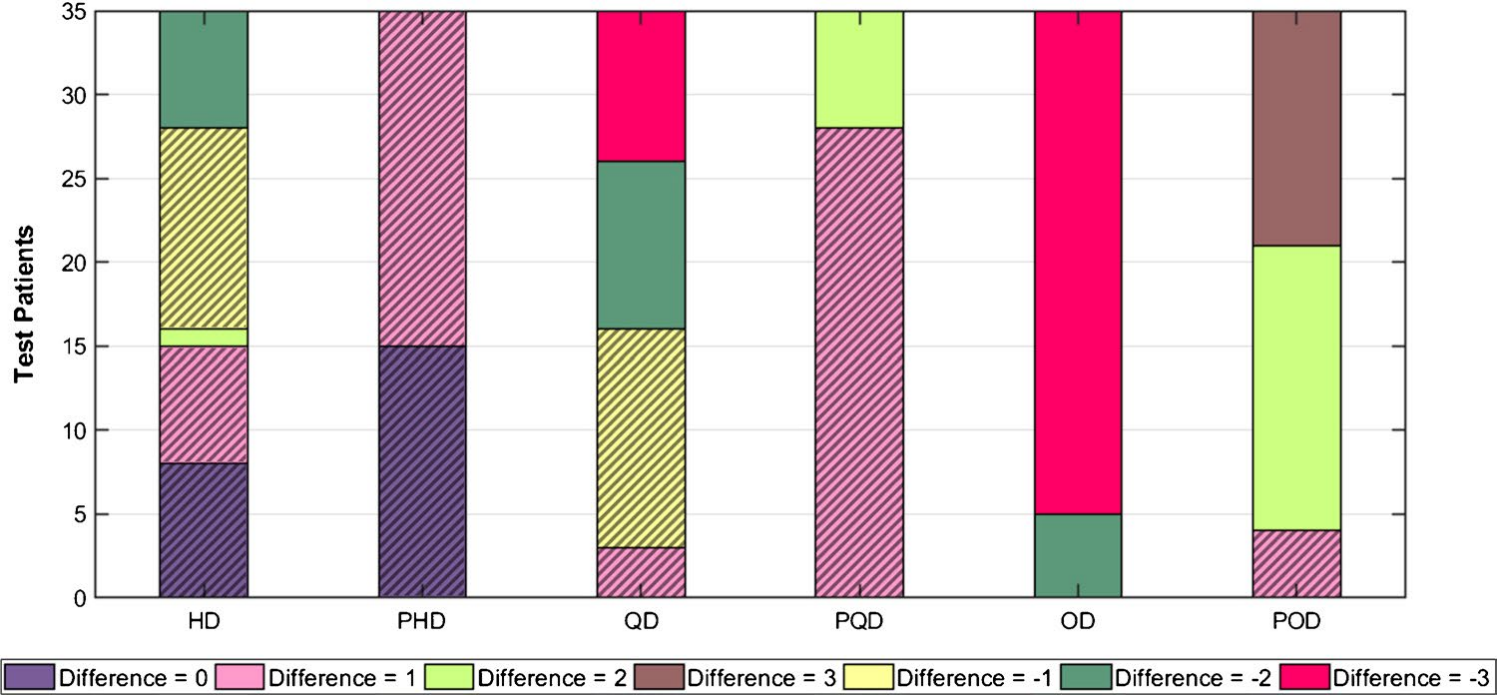
Results of image quality assessment (summed score difference) by the nuclear medicine specialist for the low-dose and predicted standard-dose images at the three reduced dose levels. Clinically acceptable cases are hatched. HD, half-dose, PHD, predicted half-dose, QD, quarter-dose, PQD, predicted quarter-dose, OD, one-eighth-dose, POD, predicted one-eighth-dose

The summed scores assigned by the nuclear medicine physician for the low-dose, standard-dose, and predicted standard-dose images, as well as diagnostic changes compared with the reference standard-dose, are presented in Supplemental Table 1 for all patients in the external test dataset. Pearson correlation coefficients (Table 4) and bar plots (Fig. 8) were employed to summarize the information in Supplemental Table 1. The Pearson correlation coefficients increased considerably for the scores assigned to the predicted standard-dose images compared to the corresponding low-dose images at the three dose levels. However, the prediction from one-eighth-dose exhibited less significant correlation coefficient (86%). We carried out a paired sample *t*-test at 5% significance level on the SS values distributions for the low-dose and the corresponding predicted standard-dose values. The *t*-test resulted in *p* values < 0.001, indicating that the mean values for the low-dose and predicted standard-dose data were statistically different.

**Table 4:** Pearson correlation coefficients for SS values assigned by the nuclear medicine specialist. The *p* value between the low-dose and the predicted standard-dose projections at each reduced dose level are given

Parameter	HD	PHD	QD	PQD	OD	POD
**Pearson correlation coefficient**	0.909	0.984	0.823	0.963	0.665	0.861
	*p* value < 0.001		*p* value < 0.001		*p* value < 0.001	

*HD* half-dose, *PHD* predicted half-dose, *QD* quarter-dose, *PQD* predicted quarter-dose, *OD* one-eighth-dose, *POD* predicted one-eighth-dose

A bar chart was used to display the diagnostic differences in the low-dose and predicted standard-dose images compared to reference images. Figure 8 shows that the absolute value of the differences decreased from low-dose to predicted standard-dose at all three reduced dose levels. The physician’s assessment enabled to conclude that cases with a score difference of 0 and ± 1 were considered clinically acceptable with no notable diagnostic changes which are indicated by hatched charts in Fig. 8. In this light, the percentage of acceptable cases was 100% for the half-dose, 80% for the quarter-dose, and 11% for the quarter-dose level. To provide a more comprehensive assessment of the model based on patients’ gender, bar plots of the performance differences for 20 female and 15 male subjects are presented in Supplemental Figs. 14 and 15, respectively. The percentage of acceptable cases is almost equal to gender-independent analysis. Hence, there is no significant relationship between network performance and patients’ gender.

## Discussion

**Fig. 9:**
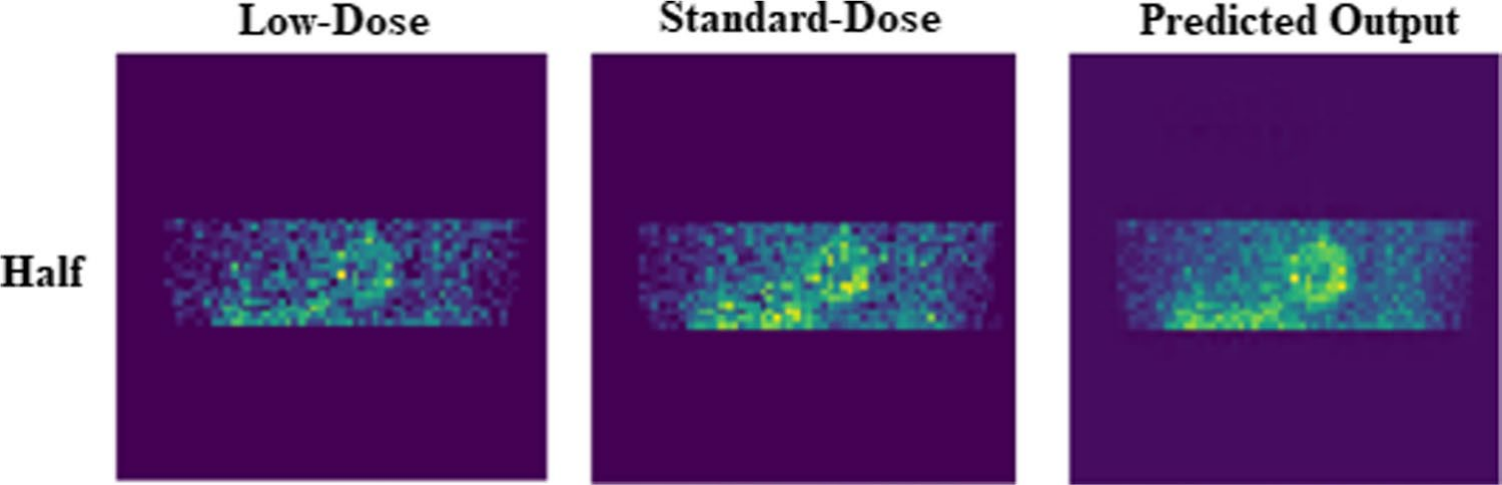
The predicted gated projections for a randomly selected patient from the test dataset at the half-dose level compared to the reference standard-dose and low-dose projections

This study aimed to assess the possibility of dose reduction in SPECT-MPI without sacrificing the diagnostic performance of the resulting images. To this end, a generative adversarial network was employed to predict high-quality standard-dose SPECT images from the corresponding low-dose data in the projection space. The proposed network was applied to suppress noise in the non-gated projections at different reduced dose levels. The clinical assessment showed that the proposed network has a promising performance for half- and quarter-dose levels. All estimated SPECT images at half-dose level were considered clinically acceptable. However, at quarter-dose level, the number of clinically acceptable cases was reduced to 80%. Evaluation at quarter-dose level revealed that almost all poor-quality predicted cases are associated with patients diagnosed with moderate/severe-risk conditions. The performance of the deep learning model for special cases largely depends on the training samples containing similar and/or representative cases. Since patients with moderate/severe medical diagnosis are less abundant in the training dataset, the performance of the network is relatively limited for these patients at quarter and one-eighth-dose levels. Supplemental Fig. 16 depicts a representative study illustrating suboptimal performance of the model for a patient with moderate risks. Adding more similar samples to the training dataset could potentially reduce the number and severity of such outliers. Dose reduction in gated SPECT-MPI was also considered. However, since the signal-to-noise ratio (SNR) in gated images is remarkably poor compared to the non-gated images, only half-dose level was studied. The fact that gated imaging was conducted in 8-time intervals, the projection data already bear high noise levels, and dose reduction by half led to extremely poor SNR. Figure 9 shows a representative predicted gated projection compared to half-dose and the reference standard-dose counterparts, wherein the excessive amount of noise in the reduced dose, as well as the reference gated projections, led to over-smoothed predictions. Dose reduction in gated SPECT-MPI faces the challenge of poor SNR and noise-induced artifacts. However, deep learning approaches (such as the proposed GAN model) could be employed to enhance the quality of standard/conventional gated SPECT-MPI (without dose reduction) as they have almost the same signal-to-noise properties of the one-eight low-dose non-gated images.

Regarding similar previous works in SPECT-MPI, Ramon et al. [30] proposed a couple of 3D convolutional auto-encoders with/without skip connections to denoise the reconstructed non-gated images (corrected for attenuation and scatter) at 1/2, 1/4, 1/8, and 1/16 of standard-dose levels. Reconstruction strategies used in this study were optimized previously for low-dose acquisitions [12]. They reported a Pearson correlation coefficient of 0.992 ± 0.001 for predicted images compared to 0.982 ± 0.001 for the low-dose input at 1/4-dose level with respect to standard-dose OSEM reconstructed images. A similar observation was made in our study wherein the Pearson correlation coefficient improved from *ρ* = 0.987 ± 0.007 to *ρ* = 0.994 ± 0.003 at the quarter-dose. It should be noted Ramon et al. performed data acquisition using a 1-day rest/stress SPECT-MPI protocol with administrated activities ranging from 1110 to 1332 MBq, while in our study, the patients underwent a 2-day rest/stress SPECT-MPI protocol with administrated activities ranging between 740 and 925 MBq. Therefore, the absolute amount of injected activity at quarter-dose level in our study is less than the administrated activity in the above-referenced study. Nevertheless, a significant/comparable improvement was observed at the quarter-dose level.

Shiri et al. [32] employed a residual deep learning model to reduce the acquisition time per projection by 50%, wherein the full-time projections were predicted from the corresponding half-time projections. The reconstruction of the predicted projections led to SSIM = 0.98 ± 0.01, PSNR = 36.0 ± 1.4, RMSE = 3.1 ± 1.1, and Pearson correlation *ρ* = 0.987 in the image domain. Shiri et al. only studied half-time acquisition in their work. Relatively better performance was observed at 1/2-dose level in our study with SSIM of 0.99 ± 0.01, PSNR of 42.49 ± 2.37, RMSE of 1.99 ± 0.63, and Pearson correlation coefficient of 0.997 ± 0.001. It should be noted that both studies used the same SPECT scanner, injected activities, and acquisition protocols. In this study, in addition to the investigation of the 1/4-dose and 1/8-dose levels, a detailed clinical assessment was performed by a nuclear medicine physician to provide a useful insight into the clinical value of the resulting images.

Though there is a fundamental difference between fast image acquisition and low-dose imaging, the reduced number of detected photons would lead to information loss and lower SNR which can significantly affect clinical diagnosis in both scenarios. However, fast image acquisition would be less affected by involuntary patient movement, such as respiratory motion. In this light, the noise suppression model investigated in this study could also be employed for fast image acquisition protocols. It is worth emphasizing that clinical adoption of low-dose SPECT-MPI could significantly reduce radiation dose to patients and hence the risks from nuclear medicine examinations to both adult and pediatric population [36].

Previous work reported on the comparison of the performance of standard-dose PET image prediction from low-dose (5%) PET in the image space versus the projection space [37]. It was concluded that standard-dose PET image estimation in the projection space exhibited more accurate/robust performance and produced higher image quality compared to implementation in image space. Moreover, network training in projection space is independent of image reconstruction algorithm and post-processing techniques. Hence, the results reported in this work would be generalizable to other clinical settings.

This study was set out to investigate the magnitude of reasonable dose reduction in SPECT- MPI without scarifying image quality. To this end, errors in clinical interpretation were considered as criteria to determine acceptable recovery of low-dose images. Though the deep learning model was capable of significantly enhancing the overall quality of high low-dose SPECT images (for 1/4 and 1/8 low-dose levels), the number of outliers with gross errors would limit the applicability of these levels in clinical setting. Special caution should be exercised to the occurrence of outliers resulting from deep learning models when ultra-low-dose imaging is sought (such as gated imaging). Deep neural networks exhibit higher robustness and performance when the task-specific training is performed using data acquired on a single SPECT camera with the same acquisition protocol. Moreover, the size of the training cohort greatly determines the robustness and overall accuracy of the model. In this regard, the training cohort should contain a realistic/balanced distribution of patients/pathologies to develop a comprehensive deep learning solution. Advances in the field provide more powerful and accurate deep learning models which would enable further dose reduction in SPECT and PET imaging. Nevertheless, task-specific data collection and creation of training datasets with a large number of samples and realistic distribution is essential for the development of robust deep learning models.

## Conclusion

This study was set out to investigate the feasibility of dose reduction in SPECT-MPI without sacrificing the quantitative accuracy and clinical value of the resulting images. A deep learning framework was assessed at different reduced dose levels to predict standard projection data from low-dose counterparts. The clinical assessment and quantitative analysis demonstrated that the deep learning model could effectively recover the underlying information in 1/2-dose and 1/4-dose SPECT images. However, due to the extremely high noise levels in 1/8-dose and gated SPECT-MPI, the deep learning model failed to fully recover the underlying signal/image quality.

## Supplementary Information

Below is the link to the electronic supplementary material.

Supplementary file1 (PDF 1407 KB)

## Abbreviations

SPECT: Single-photon emission computed tomography
MPI: Myocardial perfusion imaging
Cn: Convolutional neural network
GAN: Generative adversarial network
SD: Standard-dose
HD/PHD: Half-dose/predicted half-dose
QD/PQD: Quarter-dose/predicted quarter-dose
OD/POD: One-eighth-dose/predicted one-eighth-dose
SS: Summed score
