# Supplementary material for: Deep learning–based denoising of low-dose SPECT myocardial perfusion images: quantitative assessment and clinical performance
Source: Eur J Nucl Med Mol Imaging. 2021 Nov 15;49(5):1508–22. doi: 10.1007/s00259-021-05614-7 (PMC8940834; doi:10.1007/s00259-021-05614-7)
Supplement: Supplementary file 1 — Supplementary file1 (PDF 1407 KB) [file 259_2021_5614_MOESM1_ESM.pdf]

Supplemental Table 1. Summed score (SS) values assigned by the nuclear medicine specialist for 35 patients in the test dataset for the non-gated standard, half, quarter, and one-eighth dose levels. Difference (Dif) values show the diagnostic changes in the low-dose/predicted standard-dose images compared to the reference standard-dose data at the different dose levels.

Patients	SD	HD		PHD		QD		PQD		OD		POD	
	SS	SS	Dif	SS	Dif	SS	Dif	SS	Dif	SS	Dif	SS	Dif
**1**	6	7	-1	6	0	10	-2	3	+1	15	-3	0	+2
**2**	18	22	-2	15	+1	32	-3	12	+2	48	-3	9	+3
**3**	14	16	-1	12	+1	20	-3	9	+2	22	-3	7	+2
**4**	3	8	-2	3	0	16	-3	2	+1	30	-3	0	+2
**5**	1	2	-1	0	+1	2	-1	0	+1	6	-2	0	+1
**6**	3	4	-1	2	+1	8	-2	2	+1	6	-2	0	+3
**7**	7	5	+1	5	+1	11	-2	5	+1	24	-3	1	+3
**8**	5	6	-1	5	0	8	-1	4	+1	14	-3	2	+3
**9**	23	22	+1	20	+1	28	-2	17	+2	41	-3	11	+3
**10**	5	3	+1	4	+1	11	-3	4	+1	21	-3	2	+2
**11**	6	6	0	6	0	8	-1	5	+1	22	-3	2	+2
**12**	12	8	+2	11	+1	10	+1	7	+2	25	-3	3	+3
**13**	14	20	-2	12	+1	22	-3	10	+2	26	-3	2	+3
**14**	3	3	0	3	0	8	-2	2	+1	14	-3	0	+2
**15**	4	4	0	4	0	6	-1	3	+1	13	-3	1	+3
**16**	4	3	+1	4	0	8	-1	1	+2	22	-3	0	+3
**17**	4	4	0	4	+1	6	-1	3	+1	12	-2	1	+3
**18**	8	6	+1	6	+1	6	+1	3	+2	32	-3	6	+1
**19**	4	4	0	3	+1	6	-1	2	+1	18	-3	1	+2
**20**	3	9	-2	3	0	11	-3	2	+1	26	-3	0	+2
**21**	10	12	-1	8	+1	19	-3	7	+1	38	-3	4	+2
**22**	4	6	-1	3	+1	12	-3	3	+1	24	-3	1	+2
**23**	8	12	-2	7	+1	18	-3	6	+1	32	-3	3	+3
**24**	4	5	-1	4	0	6	-1	3	+1	18	-3	1	+2
**25**	5	4	+1	3	+1	7	-1	4	+1	22	-3	1	+2
**26**	4	4	0	3	+1	6	-1	3	+1	14	-3	2	+2
**27**	12	14	-1	10	+1	16	-2	9	+1	33	-3	4	+3
**28**	7	8	-1	5	+1	14	-2	5	+1	35	-3	3	+2
**29**	3	3	0	3	0	2	+1	2	+1	6	-2	1	+2
**30**	4	3	+1	4	0	6	-1	3	+1	16	-3	1	+2
**31**	2	4	-1	0	+1	8	-2	0	+1	14	-3	1	+1
**32**	7	11	-2	7	0	12	-2	5	+1	25	-3	3	+2
**33**	2	6	-2	2	0	14	-2	1	+1	28	-3	1	+1
**34**	14	15	-1	14	0	16	-1	12	+1	21	-2	6	+3
**35**	11	11	0	11	0	14	-1	8	+1	22	-3	6	+3

SD: Standard-Dose, HD: Half-Dose, PHD: Predicted Half-Dose, QD: Quarter-Dose, PQD: Predicted Quarter-Dose, OD: One-eighth-Dose, POD: Predicted One-eighth-Dose.

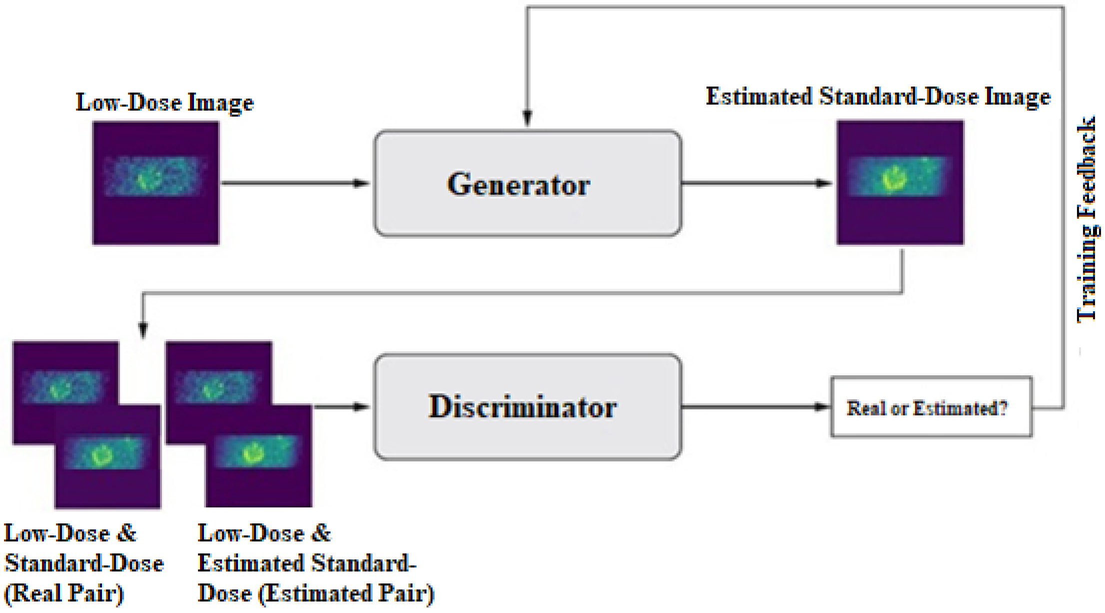

Supplemental Figure 1. Schematic illustration of the implemented GAN model.

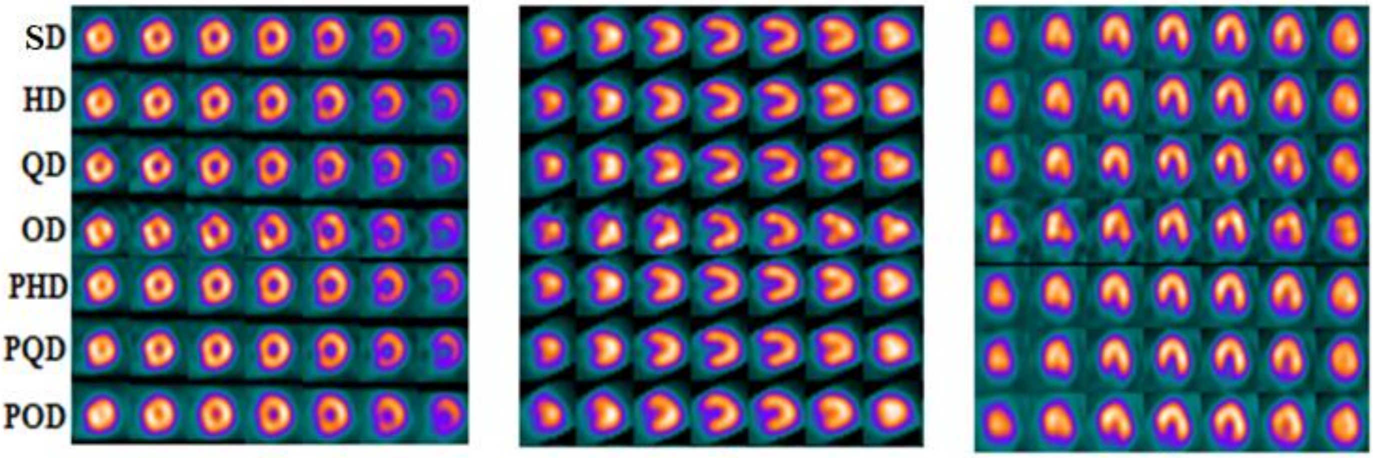

(a) (b) (c)

Supplemental Figure 2. Reconstructed non-gated images for a patient diagnosed with normal perfusion. (a): short-axis view, (b): long vertical-axis view, (c): horizontal long-axis view. In (a), (b), and (c), the rows from top to bottom correspond to the standard-dose (SD), half-dose (HD), quarter-dose (QD), one-eighth-dose (OD), predicted half-dose (PHD), predicted quarter-dose (PQD), and predicted one-eighth-dose (POD), respectively.

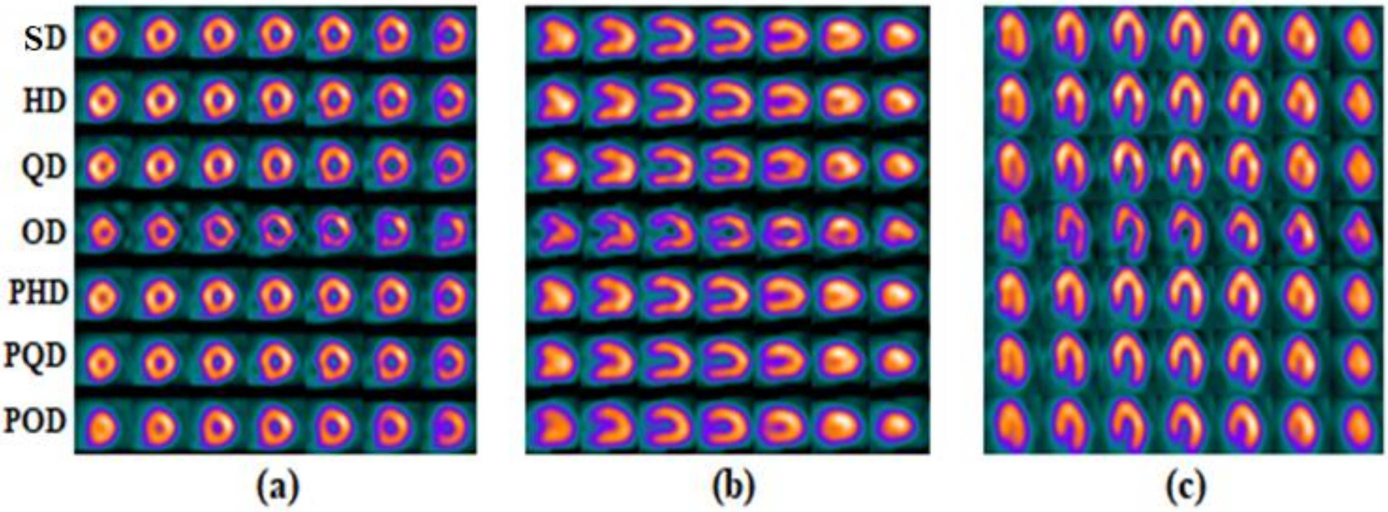

Supplemental Figure 3. Reconstructed non-gated images for a patient diagnosed with low-risk. (a): short-axis view, (b): long vertical-axis view, (c): horizontal long-axis view. In (a), (b), and (c), the rows from top to bottom correspond to the standard-dose (SD), half-dose (HD), quarter-dose (QD), one-eighth-dose (OD), predicted half-dose (PHD), predicted quarter-dose (PQD), and predicted one-eighth-dose (POD), respectively.

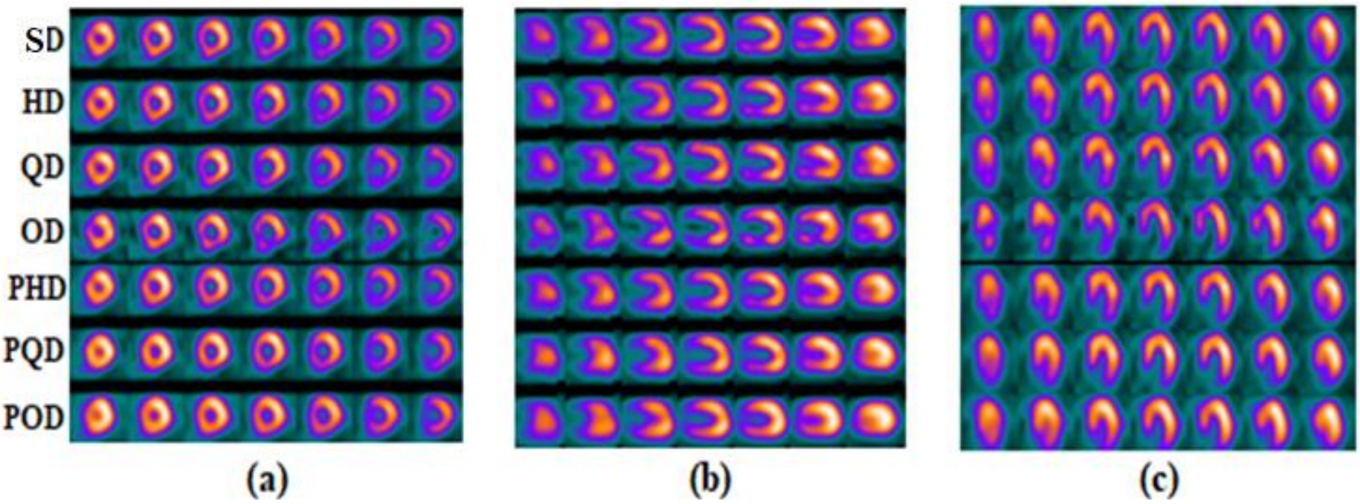

Supplemental Figure 4. Reconstructed non-gated images for a patient diagnosed with intermediate-risk. (a): short-axis view, (b): long vertical-axis view, (c): horizontal long-axis view. In (a), (b), and (c), the rows from top to bottom correspond to the standard-dose (SD), half-dose (HD), quarter-dose (QD), one-eighth-dose (OD), predicted half-dose (PHD), predicted quarter-dose (PQD), and predicted one-eighth-dose (POD), respectively.

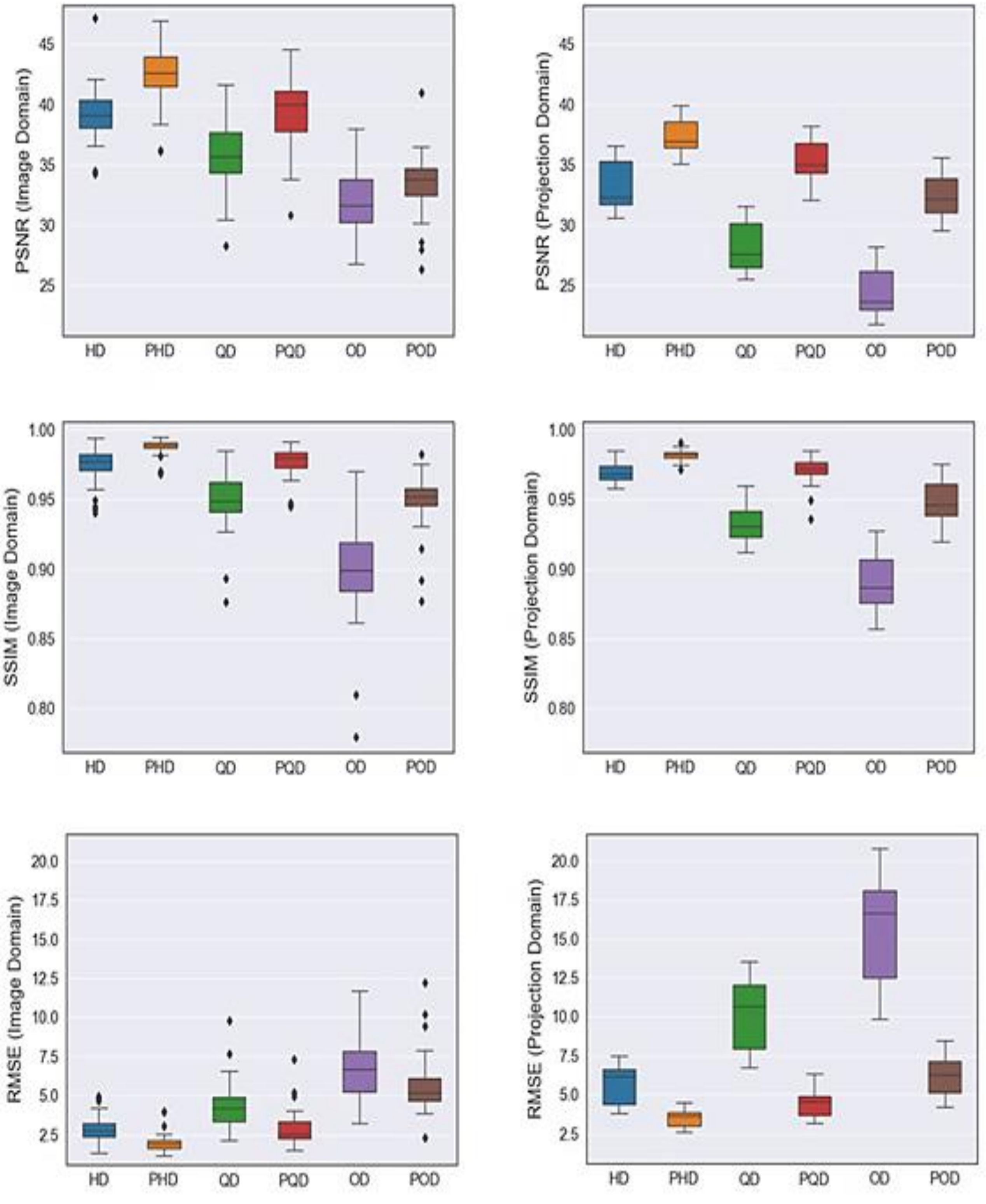

Supplemental Figure 5. Box plots comparing the quantitative parameters in the image and projection spaces. The rows from top to bottom correspond to PSNR, SSIM, and RMSE, respectively. The left and right columns show the results of the image and projection domains, respectively. HD: Half-Dose, PHD: Predicted Half-Dose, QD: Quarter-Dose, PQD: Predicted Quarter-Dose, OD: One-eighth-Dose, POD: Predicted One-eighth-Dose.

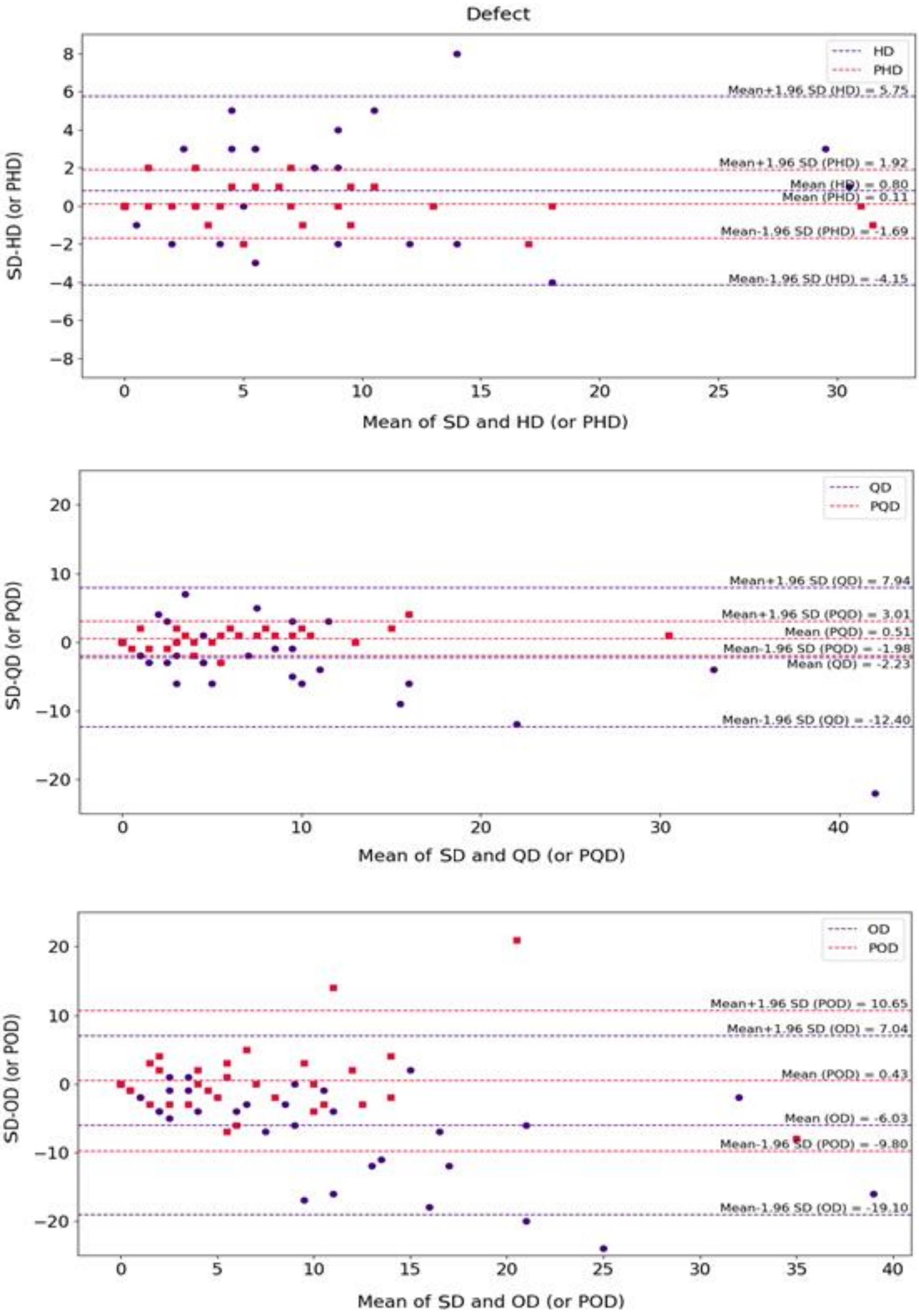

Supplemental Figure 6. Bland-Altman plots of Defect index for the low-dose and predicted standard-dose images at (a) half-dose level, (b) quarter-dose level, and (c) one-eighth-dose level compared with the reference standard-dose images. The blue and red dashed lines designate the mean and 95% confidence interval of the Defect differences in the low-dose and predicted standard-dose images, respectively. HD: Half-Dose, PHD: Predicted Half-Dose, QD: Quarter-Dose, PQD: Predicted Quarter-Dose, OD: One-eighth-Dose, POD: Predicted One-eighth-Dose.

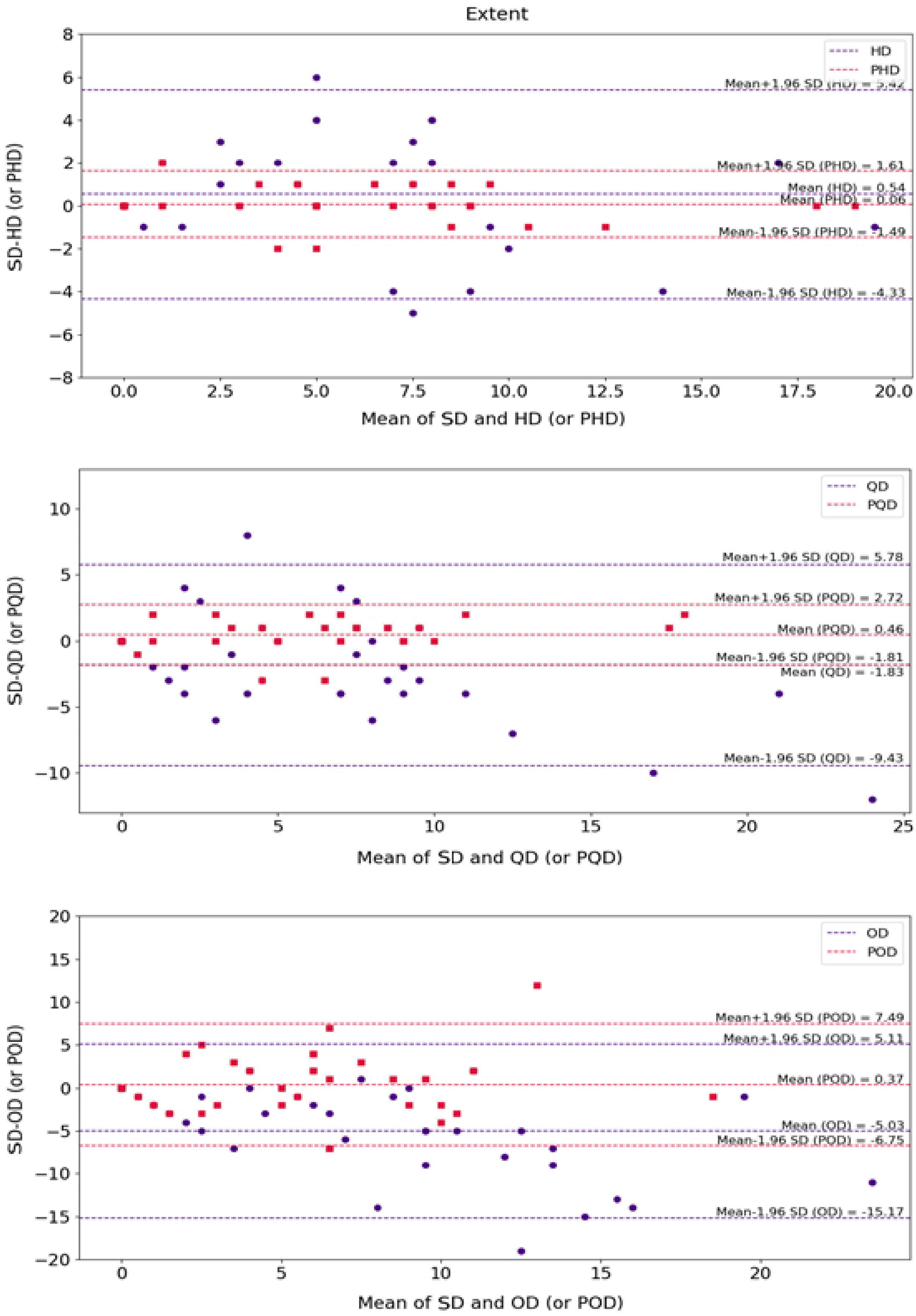

Supplemental Figure 7. Bland-Altman plots of Extent index for the low-dose and predicted standard-dose images at (a) half-dose level, (b) quarter-dose level, and (c) one-eighth-dose level compared with the reference standard-dose images. The blue and red dashed lines designate the mean and 95% confidence interval of the Extent differences in the low-dose and predicted standard-dose images, respectively. HD: Half-Dose, PHD: Predicted Half-Dose, QD: Quarter-Dose, PQD: Predicted Quarter-Dose, OD: One-eighth-Dose, POD: Predicted One-eighth-Dose.

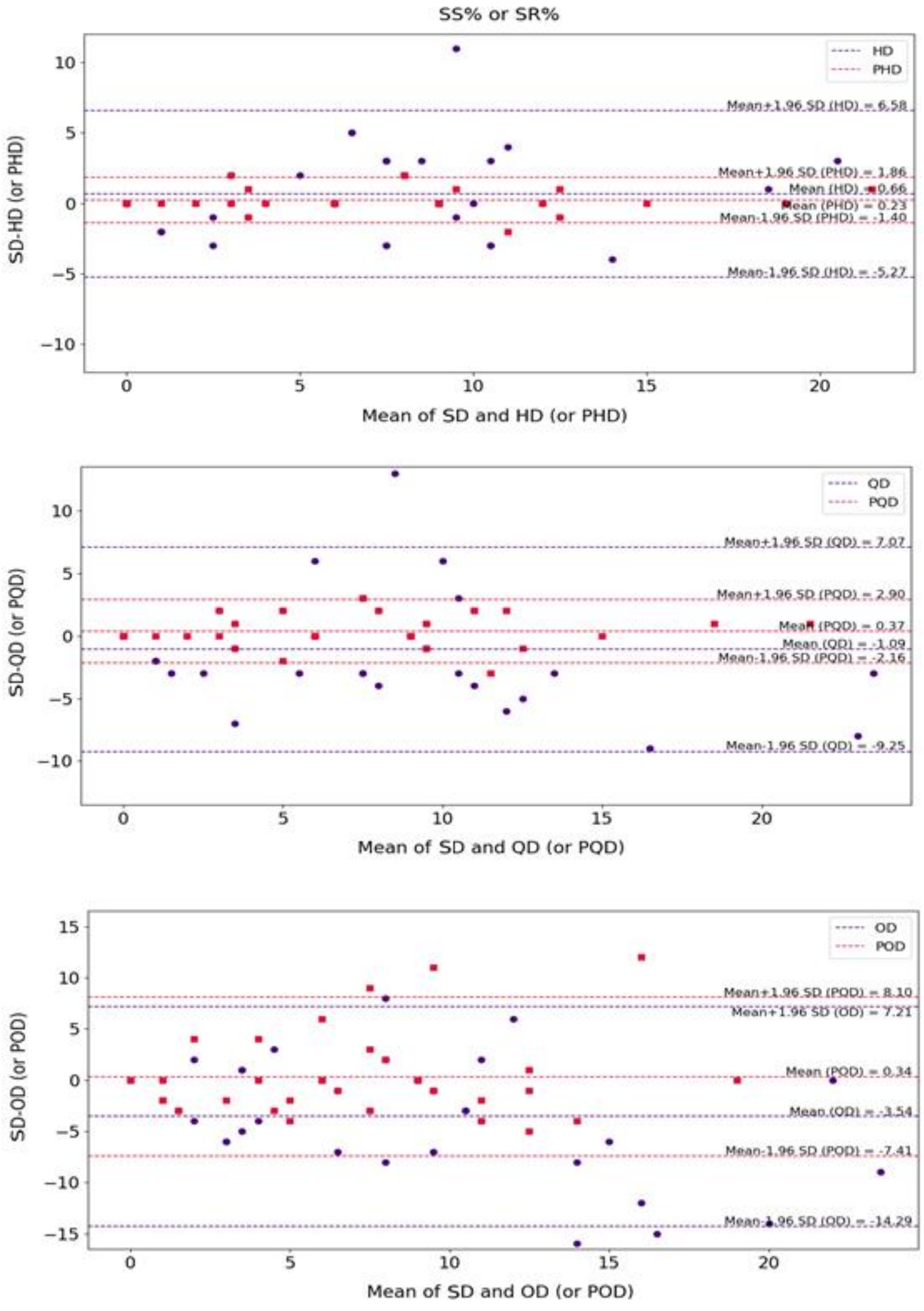

Supplemental Figure 8. Bland-Altman plots of SS% / SR% index for the low-dose and predicted standard-dose images at (a) half-dose level, (b) quarter-dose level, and (c) one-eighth-dose level compared with the reference standard-dose images. The blue and red dashed lines designate the mean and 95% confidence interval of the SS% / SR% differences in the low-dose and predicted standard-dose images, respectively. HD: Half-Dose, PHD: Predicted Half-Dose, QD: Quarter-Dose, PQD: Predicted Quarter-Dose, OD: One-eighth-Dose, POD: Predicted One-eighth-Dose.

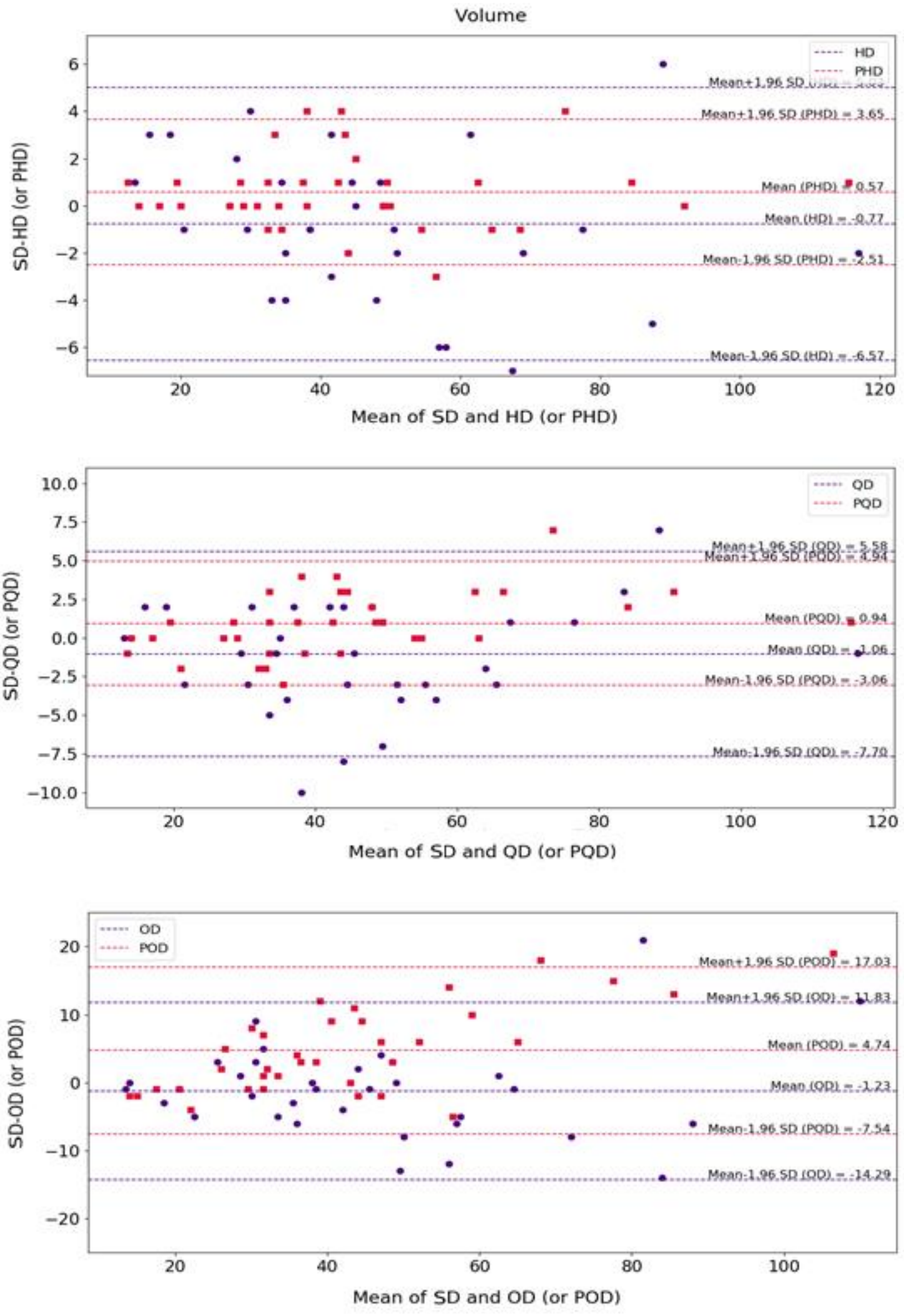

Supplemental Figure 9. Bland-Altman plots of Volume index for the low-dose and predicted standard-dose images at (a) half-dose level, (b) quarter-dose level, and (c) one-eighth-dose level compared with the reference standard-dose images. The blue and red dashed lines designate the mean and 95% confidence interval of the Volume differences in the low-dose and predicted standard-dose images, respectively. HD: Half-Dose, PHD: Predicted Half-Dose, QD: Quarter-Dose, PQD: Predicted Quarter-Dose, OD: One-eighth-Dose, POD: Predicted One-eighth-Dose.

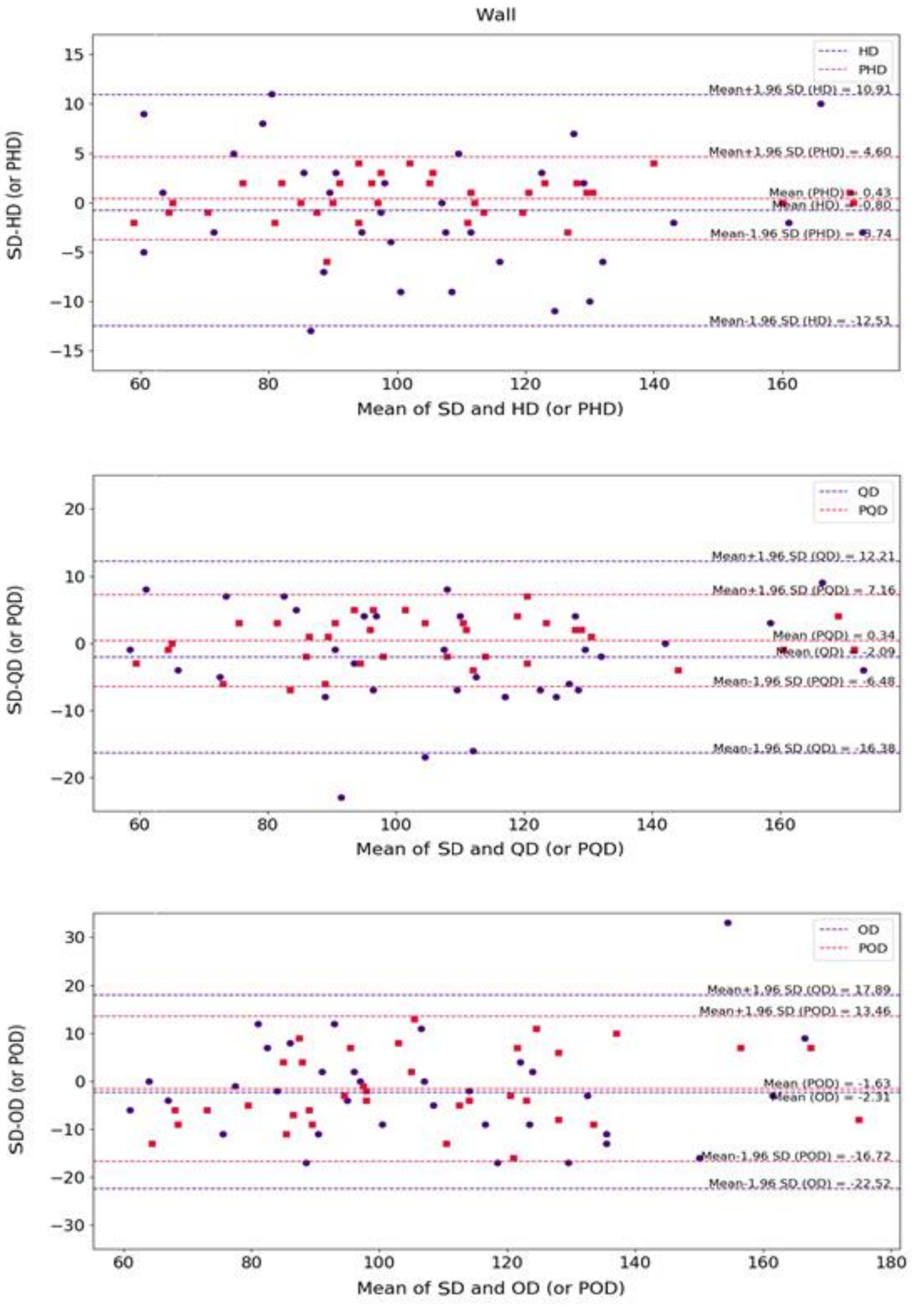

Supplemental Figure 10. Bland-Altman plots of Wall index for the low-dose and predicted standard-dose images at (a) half-dose level, (b) quarter-dose level, and (c) one-eighth-dose level compared with the reference standard-dose images. The blue and red dashed lines designate the mean and 95% confidence interval of the Wall differences in the low-dose and predicted standard-dose images, respectively. HD: Half-Dose, PHD: Predicted Half-Dose, QD: Quarter-Dose, PQD: Predicted Quarter-Dose, OD: One-eighth-Dose, POD: Predicted One-eighth-Dose.

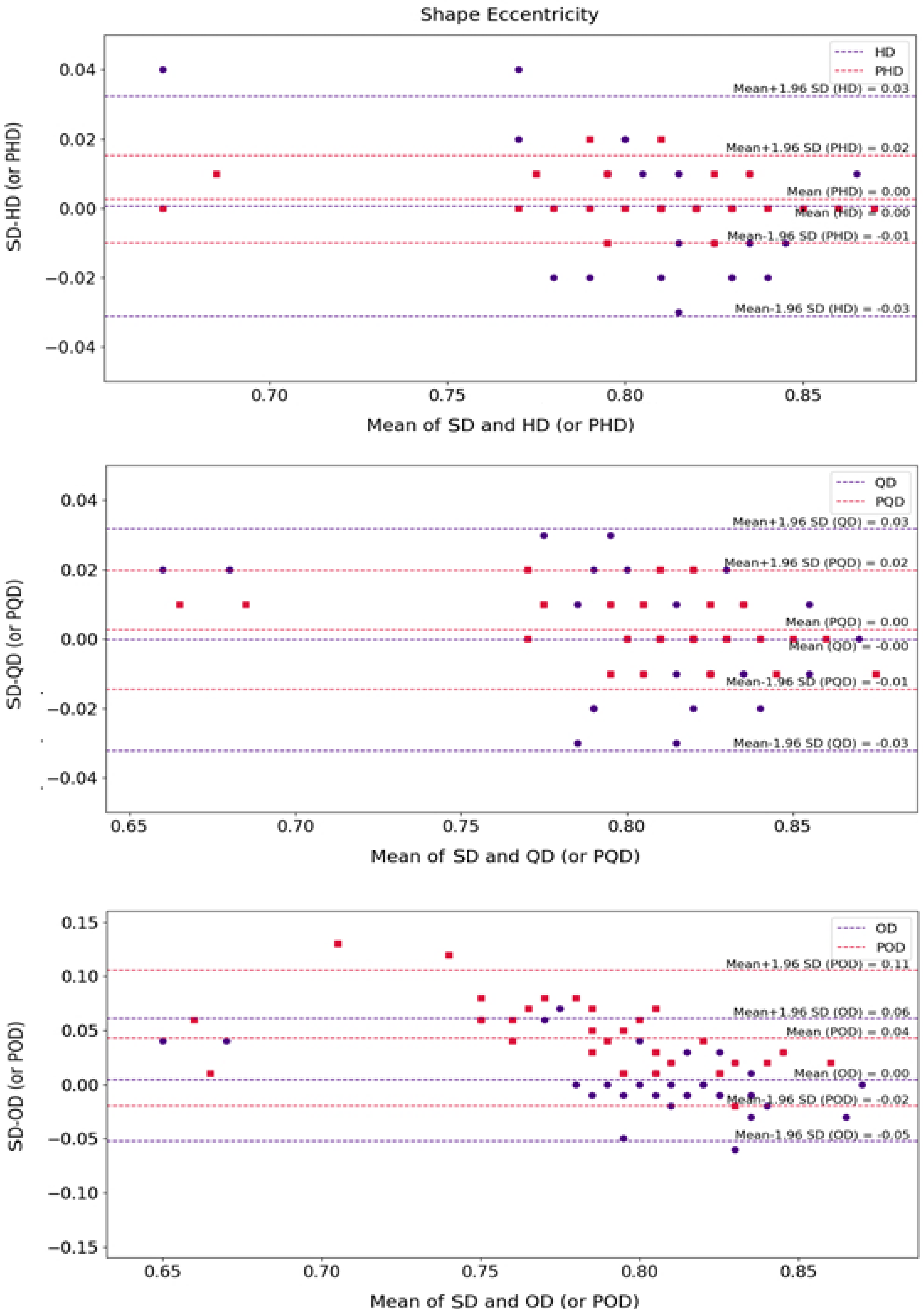

Supplemental Figure 11. Bland-Altman plots of Shape Eccentricity index for the low-dose and predicted standard-dose images at (a) half-dose level, (b) quarter-dose level, and (c) one-eighth-dose level compared with the reference standard-dose images. The blue and red dashed lines designate the mean and 95% confidence interval of the Shape Eccentricity differences in the low-dose and predicted standard-dose images, respectively. HD: Half-Dose, PHD: Predicted Half-Dose, QD: Quarter-Dose, PQD: Predicted Quarter-Dose, OD: One-eighth-Dose, POD: Predicted One-eighth-Dose.

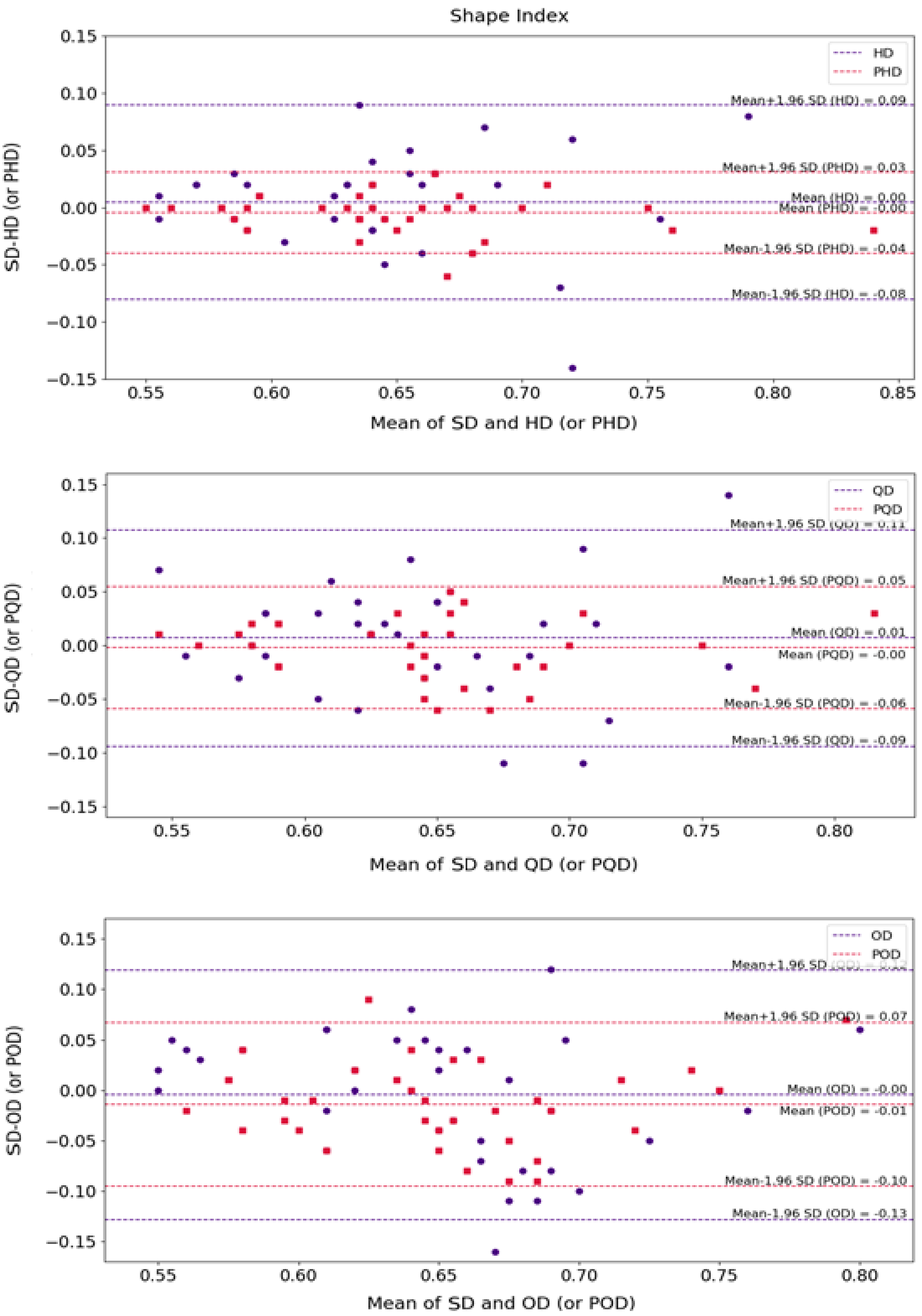

Supplemental Figure 12. Bland-Altman plots of Shape Index for the low-dose and predicted standard-dose images at (a) half-dose level, (b) quarter-dose level, and (c) one-eighth-dose level compared with the reference standard-dose images. The blue and red dashed lines designate the mean and 95% confidence interval of the Shape Index differences in the low-dose and predicted standard-dose images, respectively. HD: Half-Dose, PHD: Predicted Half-Dose, QD: Quarter-Dose, PQD: Predicted Quarter-Dose, OD: One-eighth-Dose, POD: Predicted One-eighth-Dose.

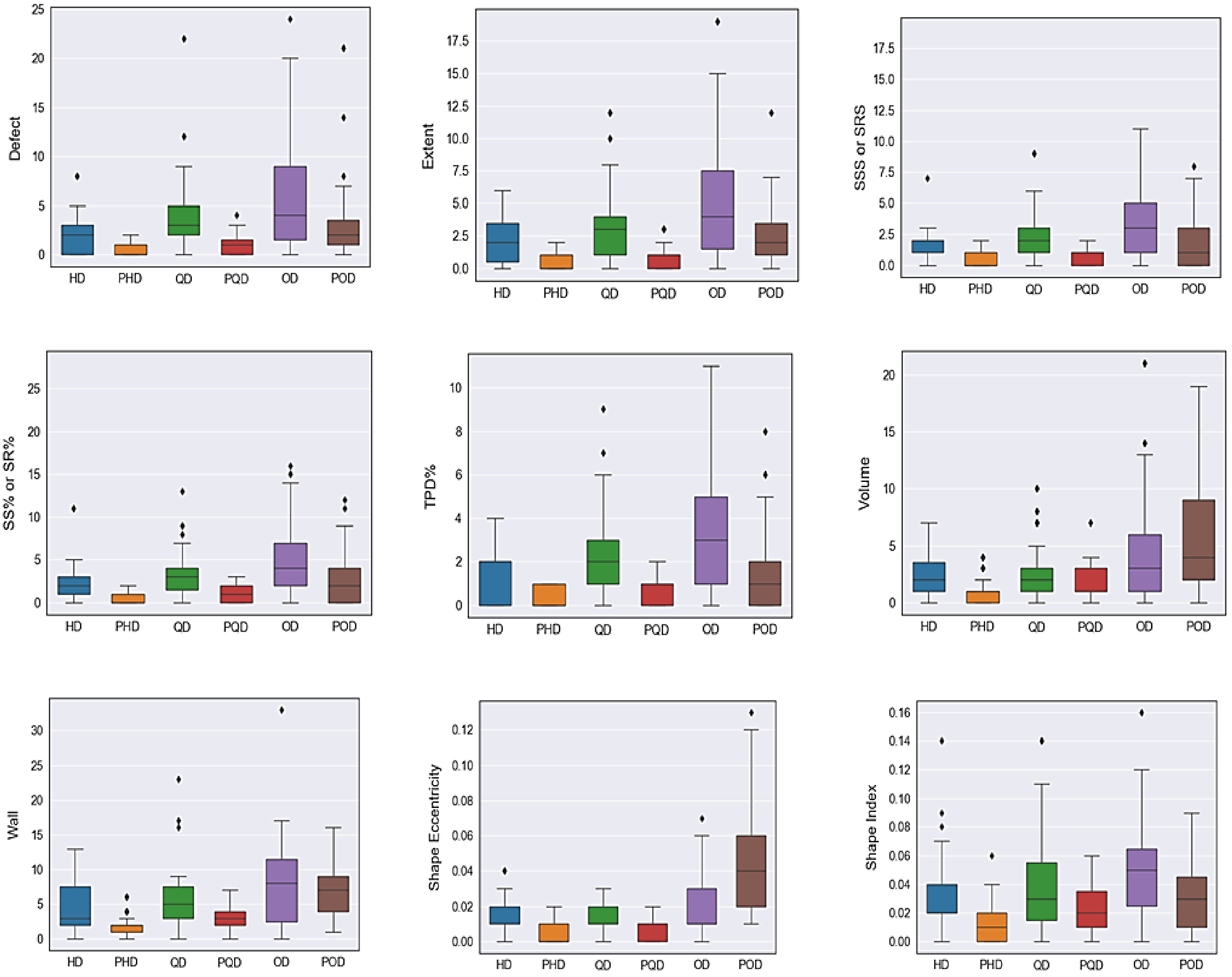

Supplemental Figure 13. The box plots of the Defect, Extent, SS% or SR%, SSS or SRS, TPD%, Volume, Wall, Shape Eccentricity, and Shape Index. Absolute differences between the low-dose/predicted standard-dose images and the reference standard-dose data at half-dose, quarter-dose, and one-eighth-dose levels. HD: Half-Dose, PHD: Predicted Half-Dose, QD: Quarter-Dose, PQD: Predicted Quarter-Dose, OD: One-eighth-Dose, POD: Predicted One-eighth-Dose.

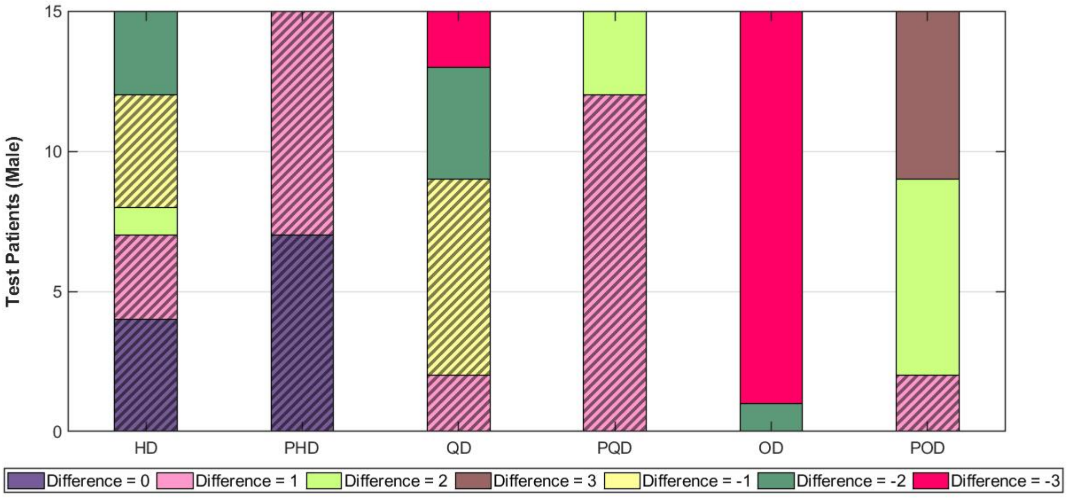

Supplemental Figure 14. Results of image quality assessment (summed score difference) for male subjects performed by the nuclear medicine specialist for the low-dose and predicted standard-dose images at the three reduced dose levels. Clinically acceptable cases are hatched. HD: Half-Dose, PHD: Predicted Half-Dose, QD: Quarter-Dose, PQD: Predicted Quarter-Dose, OD: One-eighth-Dose, POD: Predicted One-eighth-Dose.

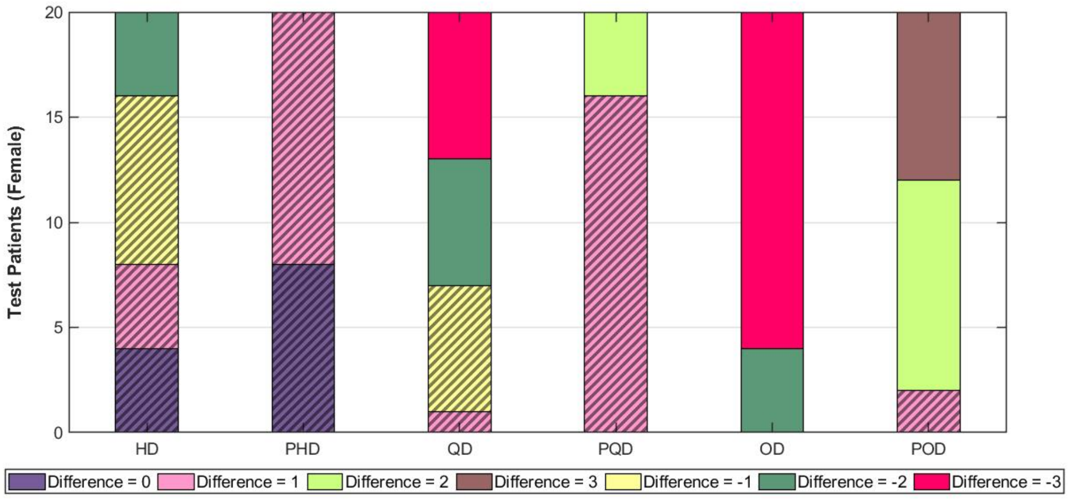

Supplemental Figure 15. Results of image quality assessment (summed score difference) for female subjects performed by the nuclear medicine specialist for the low-dose and predicted standard-dose images at the three reduced dose levels. Clinically acceptable cases are hatched. HD: Half-Dose, PHD: Predicted Half-Dose, QD: Quarter-Dose, PQD: Predicted Quarter-Dose, OD: One-eighth-Dose, POD: Predicted One-eighth-Dose.

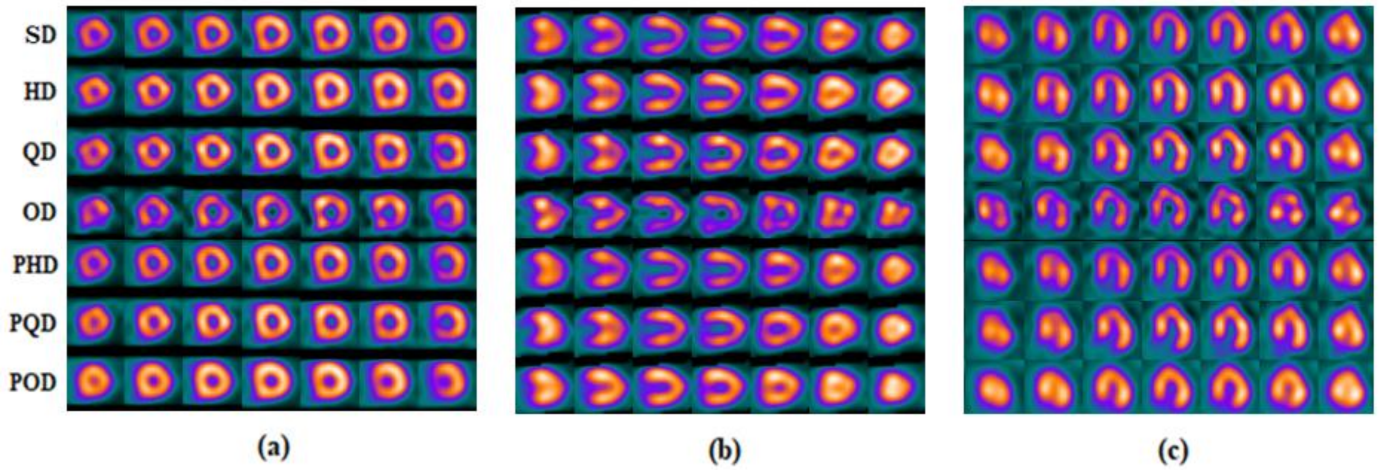

Supplemental Figure 16. Suboptimal results obtained for a patient with moderate-risks: (a) Short-axis view, (b) long vertical-axis view, and (c) horizontal long-axis view. In (a), (b), and (c), the rows from top to bottom correspond to the standard-dose (SD), half-dose (HD), quarter-dose (QD), one-eighth-dose (OD), predicted half-dose (PHD), predicted quarter-dose (PQD), and predicted one-eighth-dose (POD), respectively.
